# Inhibition of pancreatic cancer growth by an siRNA targeting *SNORA22* and a folic acid-conjugated cationic oligopeptide

**DOI:** 10.1007/s13577-026-01458-1

**Published:** 2026-09-30

**Authors:** Kazuhiro Hiyama, Miki Nishigawa, Kazunori Takagi, Kazuki Sato, Rintaro Iwata Hara, Kaoru Furihata, Kazushige Uchida, Takeshi Wada, Keisuke Taniuchi

**Affiliations:** 1 Department of Gastroenterology and Hepatology, Kochi Medical School, Kochi University, Nankoku, Kochi 783-8505 Japan; 2 Faculty of Pharmaceutical Sciences, Tokyo University of Science, Katsushika-ku, Tokyo, 125-8585 Japan; 3 NucleoTIDE and PepTIDE Drug Discovery Center, Institute of Integrated Research, Institute of Science Tokyo, Bunkyo-ku, Tokyo, 113-8519 Japan; 4 Department of Pathology, Kochi Medical School, Kochi University, Nankoku, Kochi 783-8505 Japan

**Keywords:** Pancreatic cancer, Nucleic acid compound, Small nucleolar RNA, Tumor growth, Invasion

## Abstract

The development of effective small interfering RNA (siRNA) therapeutics for pancreatic ductal adenocarcinoma (PDAC) is severely hindered by formidable physiological barriers restricting systemic stability. To address these critical challenges, we developed an advanced actively targeted drug delivery system (DDS) utilizing a novel regioisomer of a folic acid-conjugated l-2,4-diaminobutyric acid octamer (FA-Dab8α) complexed with strategically chemically modified siRNA targeting the oncogenic small nucleolar RNA *SNORA22*. Although *SNORA22* represents a potent therapeutic target for suppressing PDAC invasion and metastasis, achieving robust in vivo efficacy requires highly optimized delivery vehicles. In this study, we systematically evaluated the structural impacts of ligand conjugation, demonstrating that the α-regioisomer (FA-Dab8α) spontaneously optimizes polyplex packing to achieve remarkably superior cellular uptake, lysosomal trafficking, and target gene knockdown over its γ-counterpart. Furthermore, the strategic incorporation of phosphorothioate linkages and 2′-*O*-methyl modifications conferred exceptional RNase resistance, maintaining payload stability within the bloodstream. The therapeutic potential of this optimized platform was evaluated in a clinically relevant preclinical mouse model established with human PDAC cell line-derived organoids. Systemic administration of the *SNORA22* siRNA–FA–Dab8α complex resulted in profound tumor accumulation within 24 h and induced unprecedented tumor growth inhibition that significantly outperformed the clinical standard chemotherapy, gemcitabine plus paclitaxel (GP). Histopathological analysis revealed marked tumor necrosis and a drastic reduction in surrounding tissue infiltration, with zero detectable systemic toxicity in vital organs. These findings demonstrate that the precise structural optimization of ligand-modified oligopeptide carriers enables highly efficient oligonucleotide delivery, establishing the *SNORA22* siRNA–FA-Dab8α complex as a powerful and highly promising clinical strategy for refractory pancreatic cancer.

**Supplementary Information:**

The online version contains supplementary material available at https://doi.org/10.1007/s13577-026-01458-1.

## Introduction

Pancreatic ductal adenocarcinoma (PDAC) is a major cause of death from cancer, with approximately 250,000 patients dying annually worldwide, and is estimated to become the second most common cancer globally by 2030 [1]. The prognosis of PDAC is poor, with 1- and 5 year survival rates of only 20 and 6%, respectively [2]. About 50–60% patients present with metastatic or end-stage disease and 30–40% with localized unresectable disease, the prognosis of which is still poor even after definitive surgery [3–6]. Palliative chemotherapy [modified FOLFIRINOX (mFFX) or gemcitabine combined with albumin-bound paclitaxel (GnP)] represents the sole treatment option for PDAC patients with metastatic, end-stage, or postoperative recurrent disease [7, 8].

The final analysis of the GENERATE trial indicated that mFFX, with a median overall survival of 14.0 months, was unlikely to surpass the 17.1 months achieved with GnP [9]. Consequently, GnP is recommended as first-line chemotherapy for PDAC with distant metastases and postoperative recurrent diseases. Clinical trials investigated novel treatment strategies utilizing immune checkpoint inhibitors and molecular targeted therapies; however, none demonstrated the anticipated efficacy [10–14]. Since PDAC remains a disease with a poor prognosis, there is an urgent need to introduce new and more effective treatments into clinical practice.

RNA nanotechnology using synthetic small interfering RNA (siRNA) has recently emerged as a method for the delivery of highly promising new classes of drugs to treat human diseases. However, siRNA is highly anionic and does not readily diffuse across membrane barriers [15]. One approach to enhance the delivery of siRNA to the site of action is the development of a suitable delivery platform with characteristics that enable biocompatibility, a high loading capacity, the protection of siRNA during transport, and a high targeting ability [16]. Furthermore, because siRNA has no functional moiety targeted to the sites of interest and its negative charge leads to poor cellular uptake due to electrostatic repulsion between siRNA and the cell membrane [17], targeted delivery systems require a ligand–receptor pair that is specifically found in cancer cells.

Folic acid (FA), a synthetic monoglutamate form of folate, has been widely used as a ligand conjugate in various cancer targeting materials [18, 19]. We previously used FA-modified polyethylene glycol (PEG)–chitosan oligosaccharide lactate (COL) nanoparticles (a type of nanoparticle designed to deliver siRNA) [20]. However, the preparation of PEG–COL nanoparticles with siRNA requires complex steps that take a long time and the harvest is poor. To enhance the anti-cancer effects of oligonucleotide therapeutics against cancer cells more specifically and effectively, we have been investigating the application of DDS.

We focused on a novel cationic oligopeptide, l−2,4-diaminobutyric acid octamer (Dab8), which binds to and stabilizes siRNAs [21, 22]. Since the distance of the protonated amino groups of Dab8 corresponds to the width of a major groove in the A-type oligonucleotide duplex, such as an siRNA, Dab8 is considered to effectively interact with the anionic phosphate groups of the major groove in the A-type duplex. Therefore, we hypothesized that cancer cells effectively and specifically uptake the complex of FA-conjugated Dab8 and siRNA.

We previously reported the packaging of KH-type splicing regulatory protein (KHSRP) and its target small nucleolar RNAs (snoRNAs) into the P-bodies of the RNA granules of PDAC cells, which contributed to their invasiveness and metastasis [23]. siRNA-FA-PEG-COL nanoparticles against the specific KHSRP-bound snoRNAs, *SNORA18* and *SNORA22*, inhibited the regional invasion of the retroperitoneum and peritoneal dissemination in an orthotopic murine model of PDAC [20]. In the present study, we focused on *SNORA22*, which exerted the strongest effects against retroperitoneal invasion and lung metastasis [23]. Using the human PDAC cell line S2-013, we established a novel preclinical mouse model based on pancreatic cancer organoid culture methods [24]. This model yields tumor xenografts that closely resemble the morphology and architecture of human PDAC tissue. We utilized this preclinical model to investigate the specific uptake of SNORA22 siRNA bound to FA-Dab8 by PDAC tumors. We also evaluated the efficacy of SNORA22 siRNA bound to FA-Dab8 in inhibiting tumor growth in S2-013-organoid-derived xenograft mouse models.

## Materials and methods

### Cell culture and reagents

The human PDAC cell lines S2-013 and S2-CP8, both clonal sublines derived from the human PDAC cell line SUIT-2 [25], were utilized in this study. S2-013 is characterized as a highly invasive subline with prominent lamellipodia formation [26], whereas S2-CP8 is a distinct subline exhibiting potent liver-metastatic capacity [27]. S2-013 and S2-CP8 cells were obtained from the Cell Resource Center for Biomedical Research, Institute of Development, Aging and Cancer, Tohoku University (Sendai, Japan). The HPNE immortalized normal pancreatic epithelial cells were kindly provided as a gift by Dr. Michael Hollingsworth at the University of Nebraska (Omaha, NE). We confirmed that all cell lines were free from mycoplasma contamination using the TaKaRa PCR Mycoplasma Detection Set (Takara Bio Inc., Kusatsu, Japan) in our laboratory prior to the experiments. S2-013, S2-CP8, and HPNE cells were maintained in Dulbecco’s modified Eagle’s medium (DMEM, Gibco; Thermo Fisher Scientific, Inc., Waltham, MA, USA) containing 10% fetal calf serum (FCS) (Corning, Corning, NY, USA) as previously reported [28]. Human endothelial cells derived from human umbilical vein endothelial cells (HUVECs) and human mesenchymal stem cells (MSCs) were cultured at 37 °C as previously reported [24].

### Synthesis of siRNAs with chemical modifications

siRNAs against *SNORA18* (5′-UACUUUACUCACAGGACUATT-3′/5’-UAGUCCUGUGAGUAAAGUAAA-3’), *SNORA22* (5′-UGGCUUUGACCCUGUGCUATT-3′/5’-UAGCACAGGGUCAAAGCAAG-3’), and scrambled control siRNA (5′-UUCUCCGAACGUGUCACGUAU-3′/5’-AUACGUGACACGUUCGGAGAA-3’) were synthesized by GeneDesign Inc. (Osaka, Japan). The positions of the chemical modifications, 2′-*O*-methyl (2′-OMe) and phosphorothioate (PS), in the RNA oligonucleotides of the siRNAs targeting *SNORA18* and *SNORA22* are shown in Table 1.

**Table 1:** Sequences and chemical modifications of siRNAs

Abbreviation	
SNORA18	Sense strand: 5ʹ-r(UACUUUACUCACAGGACUA)dTdT-3ʹ Antisense strand: 5ʹ-r(UAGUCCUGUGAGUAAAGUAAA)−3ʹ
Chemically-modified SNORA18	Sense strand: 5ʹ-r(U^m^ACUUU^m^ACUC^m^AC^m^AGGACU^m^A)*dT*dT-3ʹ Antisense strand: 5ʹ-r(U^m^AGUCCUGUGAGU^m^AAAGU^m^A*A*A)−3ʹ
SNORA22	Sense strand: 5ʹ-r(UGGCUUUGACCCUGUGCUA)dTdT-3ʹ Antisense strand: 5ʹ-r(UAGCACAGGGUCAAAGCAAG)−3ʹ
Chemically-modified SNORA22	Sense strand: 5ʹ-r(UGGCUUUGACCCUGUGCU^m^A)*dT*dT-3ʹ Antisense strand: 5ʹ-r(U^m^AGC^m^AC^m^AGGGUC^m^AAAGC^m^A*A*G)−3ʹ
Scrambled control siRNA	Sense strand: 5′- UUCUCCGAACGUGUCACGUAU −3′ Antisense strand: 5ʹ-r(AUACGUGACACGUUCGGAGAA)−3ʹ

*N*^*m*^ 2′-*O*-Me modification

*Phosphorothioate modification

### Synthesis of Dab8, FA-Dab8α, and FA-Dab8γ

**Fig. 1:**
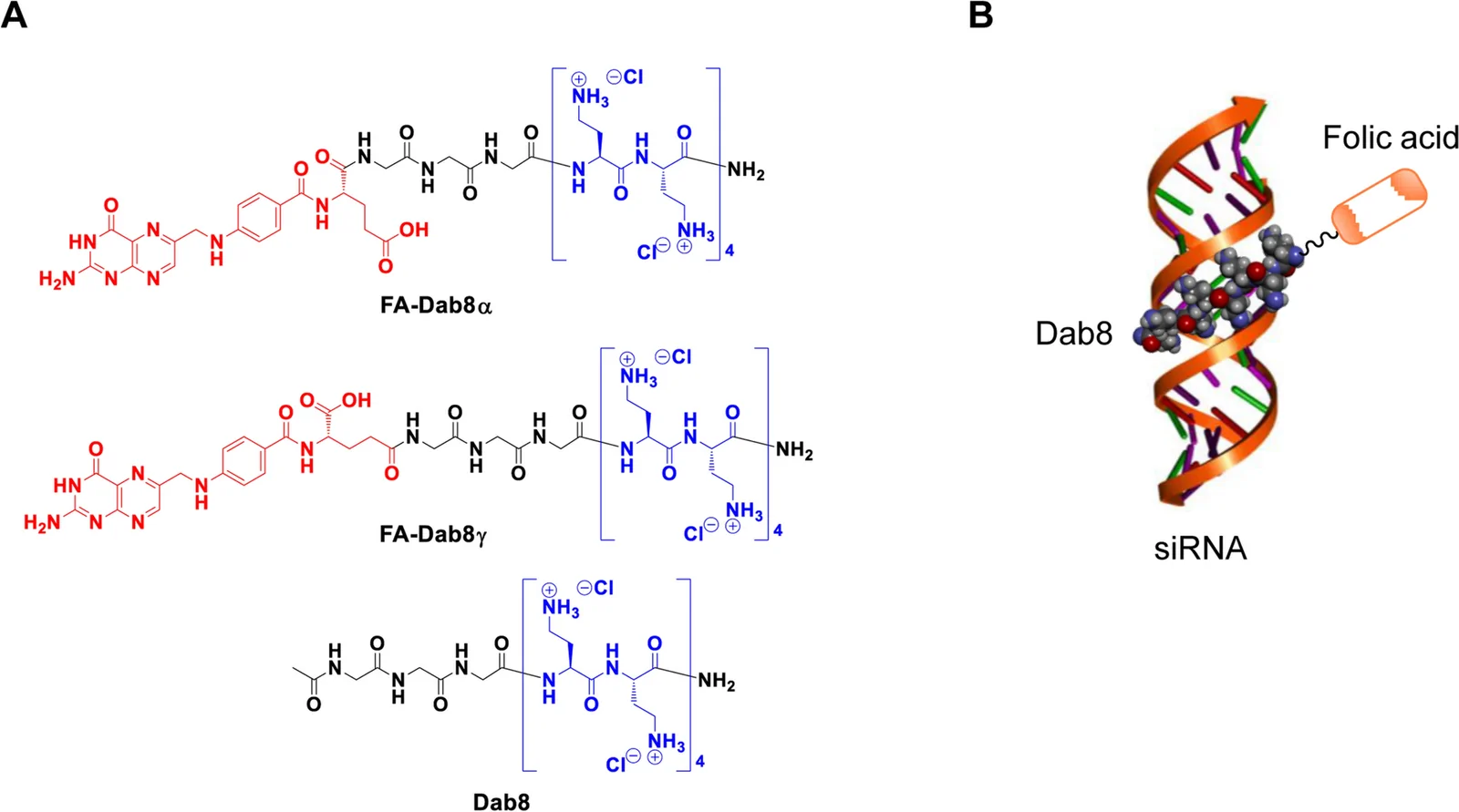
Structures of cationic oligopeptides and the siRNA-FA-Dab8 complex. **A** Structures of FA-Dab8α, FA-Dab8γ, and Dab8 in this study. **B** Schematic illustration of the siRNA-FA-Dab8 complex

All cationic peptides were used as hydrochloride salts and their structures are shown in Fig. 1. The synthesis of Dab8 was performed as previously reported [22]. Since FA-Dab8s have three consecutive Gly residues, Dab8 in the present study was designed to have a GGG sequence at the N end. The synthesis of FA-Dab8α and FA-Dab8γ was conducted by Fmoc solid-phase synthesis using Rink amide-PEG Resin XV (Watanabe Chemical Industries, Ltd.) as a solid support on a 10 μmol scale.

The cycles consisting of the removal of Fmoc and condensation were repeated as follows:

1. 25% piperidine in DMF (1 mL) was added to a reaction vessel containing the solid support to remove a Fmoc group at room temperature (rt) for 5 min.
2. A solution of 0.1 M of an amino acid monomer and HATU (0.5 mL) and a solution of 0.2 M *i*Pr_2_NEt (0.5 mL) were successively added to the reaction vessel to condense an amino group on the solid support and an amino acid monomer at rt for 15 min.

The following monomers were used for synthesis: Fmoc-Dab(Boc)-OH and Fmoc-Gly-OH. After NH_2_-GGG(Dab(Boc))_8_ was synthesized on the solid support, a solution of 0.013 M FA and 0.013 M *N,N'-*dicyclohexylcarbodiimide (DCC) in DMSO–DMF (1.3 mL, 1:1, v/v), which was pre-activated for 6 h, was added to the reaction vessel at rt and after 14 h, the solid support was washed with DMF and CHCl_3_. After the solid support was dried *in vacuo*, the deprotection and release of the product from the solid support were performed by a treatment with TFA–H_2_O–triisopropylsilane (1 mL, 96.5:2.5:1, v/v/v) at rt for 1.5 h. The solid support was filtrated, and the filtrate and washings were combined and concentrated by Ar purging. Et_2_O was added to precipitate the product, the solution was vigorously shaken and centrifuged, and the supernatant was removed. These three steps were repeated thrice, and the mixture was dried by Ar purging (supplemental Figs. S1, S2). The mixture was then analyzed and purified by reverse-phase high-performance liquid chromatography (RP-HPLC). The RP-HPLC profile indicated that two regioisomers of FA-Dab8 were obtained as a mixture because there were two main peaks (supplemental Fig. S3). The compounds corresponding to the former and latter peaks were isolated and the isolated yields were 8 and 11%, respectively. The former and later eluted isomers were hereinafter described as FA-Dab8A and FA-Dab8B for the sake of clarity.

To identify the structure of regioisomers, we synthesized FA-Dab8α as follows, referencing the study of Lainé et al. [29]*.* Using Fmoc solid phase synthesis and Rink amide-PEG Resin XV, NH_2_-GGG(Dab(Boc))_8_ was synthesized on the solid support. A solution of 0.1 M Fmoc-Glu(*t*-Bu)-OH and 0.1 M HATU (0.5 mL), and a solution of 0.2 M *i*Pr_2_NEt (0.5 mL) were successively added to the reaction vessel to introduce a Glu residue at rt for 15 min. After removal of the Fmoc group by a treatment with 25% piperidine in DMF at rt for 30 min, a solution of 0.04 M pteroic acid and 0.04 M HATU in DMF–DMSO (0.5 mL, 1:1, v/v), and that of 0.08 M *i*Pr_2_NEt in DMF–DMSO (0.5 mL, 1:1, v/v) were successively added to the reaction vessel at rt. After 15 h, the solid support was washed with DMF, and a similar procedure was then conducted to yield FA-Dab8α. The RP-HPLC analysis indicated that FA-Dab8α was eluted at 21 min, and the retention times of FA-Dab8α and FA-Dab8B were identical. Therefore, FA-Dab8A and FA-Dab8B corresponded to FA-Dab8γ and FA-Dab8α, respectively. Dab8, FA-Dab8α, and FA-Dab8γ were exchanged to hydrogen chloride salts before use.

### Quantification of FA-Dab8α and FA-Dab8γ

The quantification of FA-Dab8α and FA-Dab8γ was conducted using a UV spectrometer. Absorbance at 368 nm of the solution of FA-Dab8α or FA-Dab8γ in 1 M NaOH aq. was measured and the concentration of FA-Dab8α or FA-Dab8γ was estimated using Ɛ_368_ = 7967 L/cm/mol.

### Preparation of a complex of siRNA and Dab8, FA-Dab8α, or FA-Dab8γ

Dab8, FA-Dab8α, or FA-Dab8γ was added to a solution of siRNA, which was either a chemically modified or unmodified siRNA targeting *SNORA18*, *SNORA22*, or a scrambled control siRNA. The solution was prepared in RNase-free water at rt. The molar ratio of siRNA to Dab8 or FA-Dab8 was set to 1:1, 1:2, or 1:3. The complexes were allowed to stand at rt for 15 min.

### Serum stability assay

**Fig. 2:**
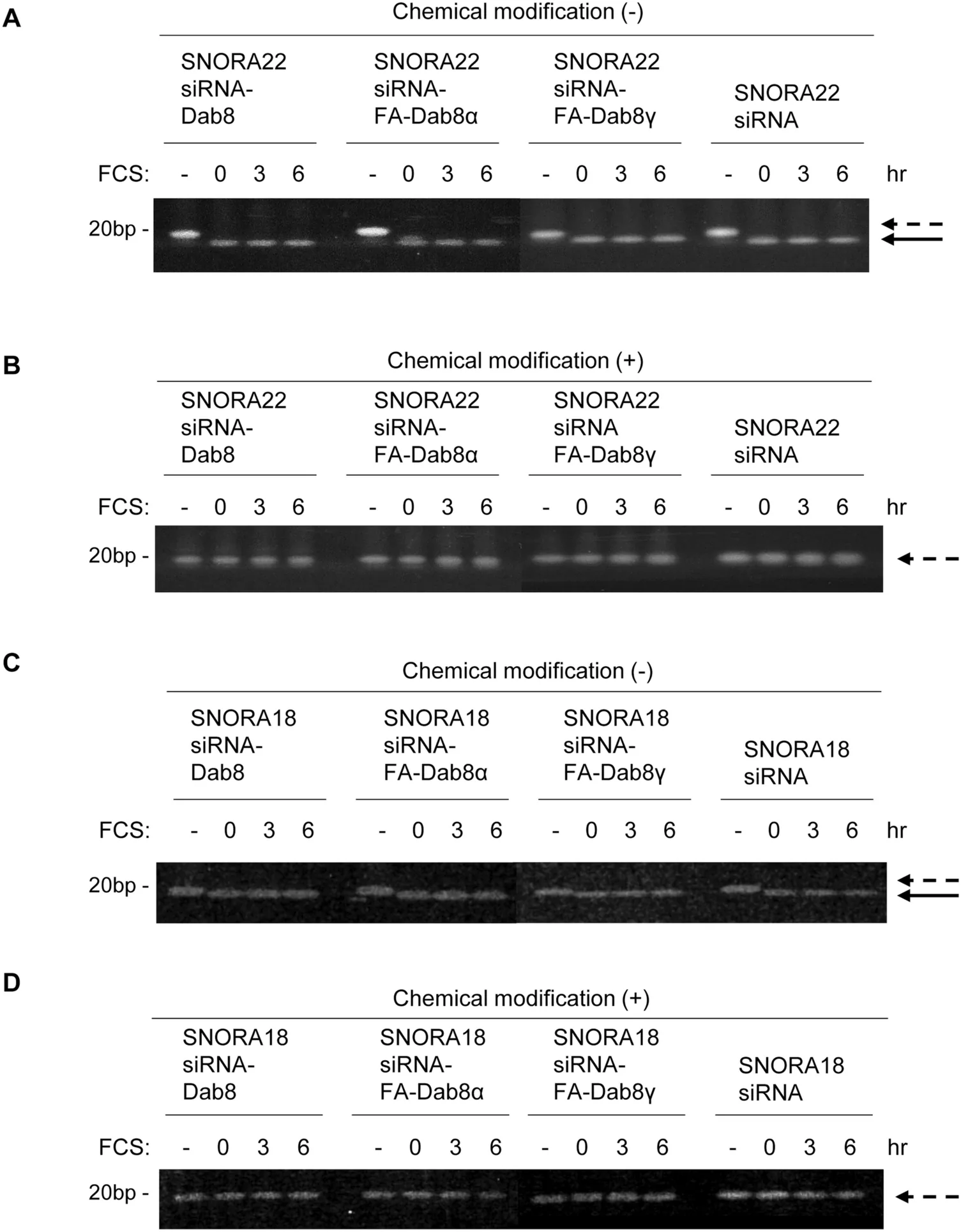
Chemical modifications to SNORA18 siRNA and SNORA22 siRNA to enhance serum stability. **A**, **C** The stability of SNORA22 siRNA in serum was compared between SNORA22 siRNA-FA-Dab8α and SNORA22 siRNA-FA-Dab8γ without chemical modifications to the siRNAs (**A**). A numerical size label (20 bp) is indicated on the left to allow verification of the molecular sizes of the detected RNA species. The dashed arrow represents undigested siRNA. The solid arrow represents digested siRNA. The same experiment was performed with *SNORA18* (**C**). **B, D** The stability of chemically modified SNORA22 siRNA in serum was compared between SNORA22 siRNA-FA-Dab8α and SNORA22 siRNA-FA-Dab8γ (**B**). A numerical size label (20 bp) is indicated as in (**A**). The dashed arrow represents undigested siRNA. The same experiment was performed with *SNORA18* (**D**)

A total of 2 pmol siRNAs in 1 μL of RNase-free water was incubated in 10 μL of PBS or 10% FCS in PBS at 37 °C for up to 6 h according to a previously reported protocol [30]. All samples were immediately flash-frozen in liquid nitrogen, and 15% native polyacrylamide gel (SuperSep DNA 15%, 17 wells, Fujifilm Wako Pure Chemical Industries, Osaka, Japan) electrophoresis was performed at 30 mA/each gel at rt for 67 min using EasySeparator (Fujifilm Wako Pure Chemical Industries, Osaka, Japan) and a Bio-Rad PowerPac HC power supply, followed by SYBR Gold staining (SYBR^™^ Gold Nucleic Acid Gel Stain, Invitrogen, Waltham, MA, USA) for 20 min. Each specimen was visualized using a BioDoc-It 220 Imaging System (UVP, Upland, CA, USA). The exposure time (UV 302 nm) was 3 s in Fig. 2.

### Confocal immunofluorescence microscopy for an uptake analysis of nucleic acid compounds

A total of 2.0×10^4^ S2-013 and 1.0×10^5^ HPNE cells/well were seeded on 4-well glass coverslips in DMEM. After 20 h, 68.2 pmol Dab8, FA-Dab8α, or FA-Dab8γ was incubated with 34.1 pmol Alexa Fluor 647-labeled scrambled control siRNA or *SNORA22* siRNA, and 10 μL of the resulting nucleic acid solution was mixed with 990 μL of DMEM. This solution was added to each well, followed by a 48-h incubation with the addition of 1 mL of fresh DMEM at 24 h. Cells were then fixed with 4% paraformaldehyde, followed by immunostaining using an anti-folate receptor 1 (FOLR1) antibody (MAB5646, R&D Systems, Minneapolis, MN, USA) and DAPI staining (HardSet Mounting Medium with DAPI, Vector Laboratories, Burlingame, CA, USA). Each specimen was visualized using a BZ-X800 microscope (Keyence, Osaka, Japan).

To investigate whether *SNORA22* siRNA released from internalized chemically modified *SNORA22* siRNA-FA-Dab8α was trafficked to lysosomes within S2-013 cells, lysosomes were stained using LysoTracker Red DND-99 (Thermo Fisher Scientific, Waltham, MA, USA). S2-013 cells were cultured on coverslips overnight, and Alexa Fluor 647-labeled, chemically modified *SNORA22* siRNA-FA-Dab8α was added to each well. After a 48-h incubation with the addition of 1 mL of fresh DMEM medium at 24 h, cells were fixed with 4% paraformaldehyde, stained with LysoTracker for 2 h according to the manufacturer’s protocol, washed with PBS, and imaged using a BZ-X800 microscope.

### Semi-quantitative reverse transcription PCR (RT-PCR)

S2-013 cells were plated in six-well plates, and chemically modified SNORA22 siRNA-Dab8, SNORA22 siRNA-FA-Dab8α, and SNORA22 siRNA-FA-Dab8γ were added to each well. Similar procedures were performed with *SNORA18*. After a 48-h incubation with the addition of fresh DMEM medium at 24 h, total RNA was extracted from each well using the RNeasy Mini Kit (Qiagen, Venlo, Netherlands) according to the manufacturer’s recommendations, treated with DNase I (Roche Applied Science, Penzberg, Germany), and quantified using a NanoDrop spectrophotometer (Thermo Fisher Scientific, Waltham, MA, USA). One microgram of total RNA was reverse transcribed into single-stranded cDNA using the SuperScript III First-Strand Synthesis System for RT-PCR (Invitrogen) and random primers. Appropriate dilutions of each single-stranded cDNA were prepared for subsequent PCR amplification, with GAPDH being monitored as a quantitative control. All reactions involved initial denaturation at 95 °C for 2 min, followed by 21 cycles (for GAPDH) or 26 cycles (for *SNORA18* and *SNORA22*) at 95 °C for 30 s, 55 °C for 30 s, and 72 °C for 1 min followed by 72 °C for 5 min on a T100 Thermal Cycler (Bio-Rad, Hercules, CA, USA).

### In vitro invasion assay

A Matrigel invasion assay was performed as previously described [31]. Briefly, 1.0×10^5^ S2-013 cells were plated in six-well plates, and chemically modified SNORA22 siRNA-Dab8, SNORA22 siRNA-FA-Dab8α, and SNORA22 siRNA-FA-Dab8γ were added to each well. Similar experiments were performed with *SNORA18*. After a 48 h incubation with the addition of fresh DMEM medium at 24 h, cell invasion was assessed using a cell invasion assay (24-well plates, membranes with a pore size of 8 µm coated with a layer of Matrigel extracellular matrix proteins; Corning Inc., Corning, NY). The assay was conducted 3 times independently.

### Electrophoretic mobility shift assay (EMSA)

EMSA was performed to assess the binding of SNORA22 siRNA to FA-Dab8α and FA-Dab8γ. Samples were prepared as described in the Serum stability assay section using a previously reported method [22, 32]. A total of 18.2 pmol of chemically modified SNORA22 siRNA and either FA-Dab8α or FA-Dab8γ were incubated in PBS or 10% FCS in PBS at 37 °C for 6 h. A 15% native polyacrylamide gel (SuperSep DNA 15%, 17 wells, Fujifilm Wako Pure Chemical Industries, Osaka, Japan) was run in a vertical electrophoresis system (Fujifilm Wako Pure Chemical Industries, Osaka, Japan) at 100 V at rt for 200 min using a Bio-Rad PowerPac HC power supply. Each gel was stained with SYBR Gold (SYBR™ Gold Nucleic Acid Gel Stain, Invitrogen, Waltham, MA, USA). Images were acquired using a BioDoc-It 220 Imaging System (UVP, Upland, CA, USA). The exposure time (UV 302 nm) was 5 s in Fig. 6A.

### Mice and xenografts

S2-013 cell line-derived organoids were established using cultured S2-013 cells, HUVECs, and MSCs as previously reported [24]. Animal experiments were approved by the Kochi University Animal Care and Use Committee (approval #R01-026) and conducted in accordance with its guidelines. To generate subcutaneous tumors, S2-013 cell line-derived organoids in 50 μL of a Matrigel matrix/DMEM mixture were subcutaneously implanted into the flanks of 7-week-old female athymic nude mice (BALB/cSlc-nu/nu) (Japan SLC, Inc., Shizuoka, Japan). After implantation, tumors were allowed to grow for one week before treatment initiation, as described below.

### Delivery of *SNORA22 siRNA*-FA-Dab8 nucleic acid compounds to xenografts in the S2-013-organoid model

Xenografts were allowed to grow for 7 weeks after the implantation of S2-013 cell line-derived organoids. Alexa Fluor 647-labeled, chemically modified *SNORA22* siRNA (5 µg) was then administered with 1, 2, or 3 equivalents of Dab8, FA-Dab8α, or FA-Dab8γ via a tail vein injection (n = 4 per group). After 24 h, fluorescence images were captured using an IVIS Spectrum In Vivo Imaging System (PerkinElmer, Waltham, MA, USA) with excitation and emission filters set at 640 and 680 nm, respectively.

### Inhibitory effects of *SNORA22* siRNA-FA-Dab8α nucleic acid compounds on the growth of xenografts in the S2-013-organoid model

Tumors were allowed to grow for 1 week after the implantation of S2-013 cell line-derived organoids. Mice were then treated once weekly with intravenous injections of chemically modified SNORA22 siRNA (5 µg) complexed with either Dab8 (2 equivalents) or FA-Dab8α (2 equivalents) (n = 8). A control group (n = 8) received scrambled control siRNA with FA-Dab8α (2 equivalents). Gemcitabine plus paclitaxel (GP) was administered at 50 mg/kg gemcitabine and 0.5 mg/kg paclitaxel on days 1, 8, and 15 of a 28 day cycle from three weeks after implantation [33]. Tumor volumes were measured weekly using calipers (n = 8 per group) and calculated using the following formula: V (mm^3^) = 0.5 × a × b^2^ (a: minor radius, b: major radius), where a represents the longest tumor diameter and b represents the perpendicular diameter. Nine (GP group) or ten (all groups other than the GP group) weeks after organoid implantation, mice were sacrificed, and solid tumors, the lungs, liver, and kidneys were collected. Tumor, lung, and liver sections were stained with hematoxylin and eosin (H&E).

### Histological analysis of organs

Following the completion of the study, mice were euthanized and their lungs, heart, liver, kidneys, adrenal glands, and spleen were collected. These organs were then sectioned and stained with H&E for a histological examination. Sections were analyzed for any abnormalities, such as inflammation or necrosis.

### Statistical analysis

StatFlex software (Ver6; YUMIT, Osaka, Japan) and SAS software (Ver9.1.3; SAS Institute, Cary, NC) were used for statistical analyses. The significance of differences between groups was assessed using the two-tailed Student’s *t*-test or Fisher’s exact test, as appropriate. In all analyses, *p* <0.05 was considered to be significant.

## Results

### Preparation of complexes of SNORA22 siRNA and FA-Dab8.

We synthesized FA-Dab8, which has two regioisomers, FA-Dab8α and FA-Dab8γ, for use in a DDS targeting PDAC cells (Fig. 1). To enhance the stability of SNORA22 siRNA in serum, we chemically modified the siRNA (Table 1). We then prepared SNORA22 siRNA-FA-Dab8α and SNORA22 siRNA-FA-Dab8γ complexes (Fig. 1B).

### Chemical modifications to SNORA22 siRNA to stabilize it in serum.

The stability of SNORA22 siRNA in serum was compared between SNORA22 siRNA-FA-Dab8α and SNORA22 siRNA-FA-Dab8γ, with and without chemical modifications. Both siRNAs without chemical modifications were degraded immediately upon mixing with serum, even in the presence of FA-Dab8α or FA-Dab8γ (Fig. 2A). In contrast, both analogs with chemical modifications remained stable for up to 6 h after mixing with serum, showing no degradation (Fig. 2B). While the addition of FA-Dab8α or FA-Dab8γ to SNORA22 siRNA did not prevent degradation, the incorporation of chemical modifications conferred serum resistance to both nucleic acid analogs. Similar results were obtained for *SNORA18*, as shown in Fig. 2C, D.

### Uptake of SNORA22 siRNA-FA-Dab8 nucleic acid compounds in PDAC and HPNE cells

**Fig. 3:**
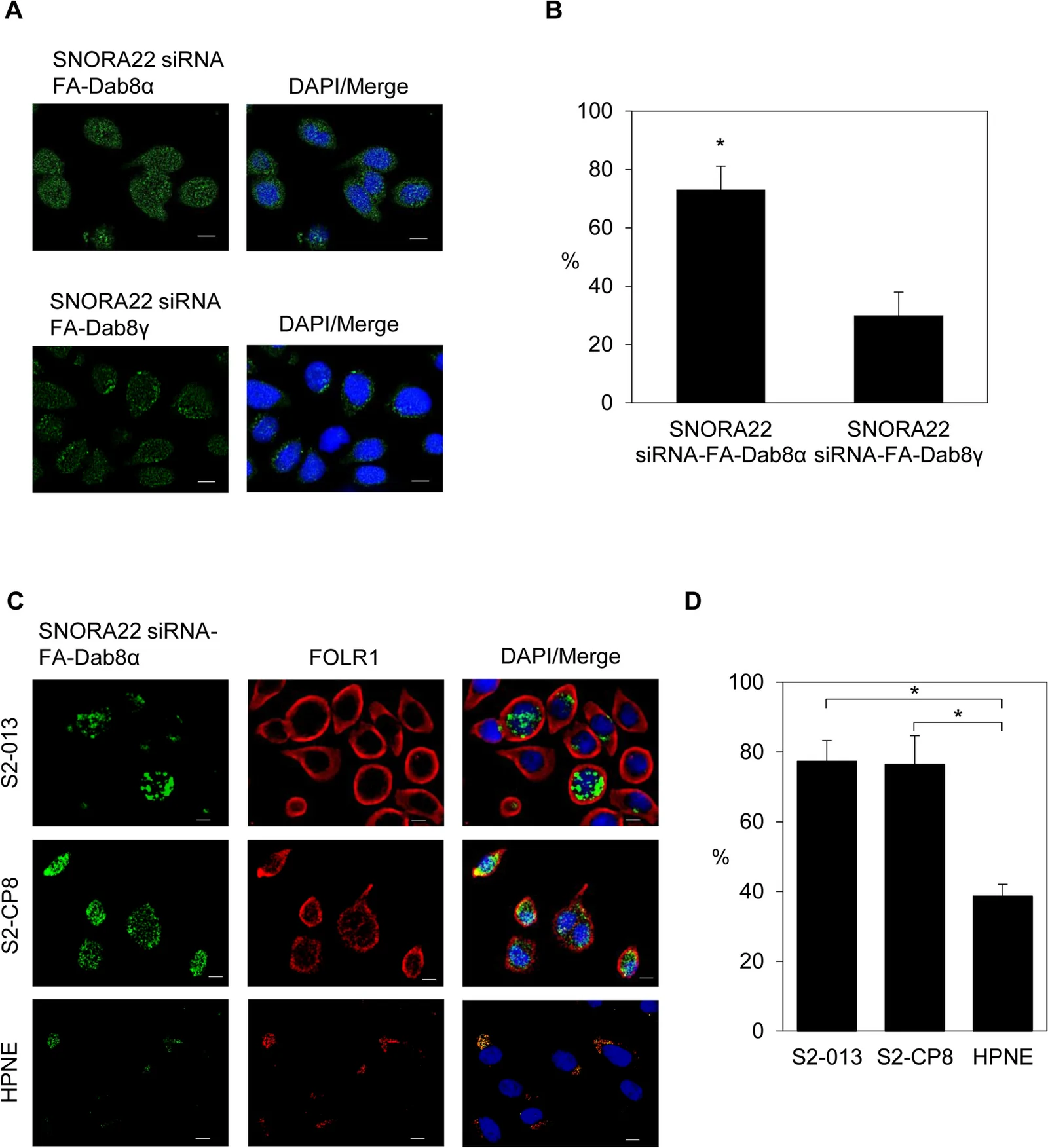
Confocal immunofluorescence images of S2-013 and HPNE cells that took up SNORA22 siRNA conjugated with FA-Dab8α and FA-Dab8γ. **A** S2-013 cells were incubated with Alexa Fluor 647-labeled chemically modified SNORA22 siRNA-FA-Dab8α and SNORA22 siRNA-FA-Dab8γ (green). Blue, DAPI staining. Scale bars, 10 µm. **B** Quantification of Alexa Fluor 647-labeled chemically modified SNORA22 siRNA-positive cells in A. Data represent the number of cells positive for the siRNA, scored across five fields per group and derived from two independent experiments. *Columns*, mean; *bars*, SD. *p <0.05 significantly different from SNORA22 siRNA-FA-Dab8γ (the Student’s *t*-test).**C** S2-013, S2-CP8, and HPNE cells were incubated with Alexa Fluor 647-labeled chemically modified SNORA22 siRNA-FA-Dab8α (green). Cells were stained with an anti-FOLR1 antibody (red). Blue, DAPI staining. Scale bars, 10 µm. **D** Quantification of Alexa Fluor 647-labeled chemically modified SNORA22 siRNA-positive cells in C. Data represent the number of cells positive for the siRNA, scored across five fields per group and derived from two independent experiments. *Columns*, mean; *bars*, SD. *p <0.05 significantly different from HPNE cells (the Student’s *t*-test)

To assess the cellular uptake of chemically modified SNORA22 siRNA-FA-Dab8α and SNORA22 siRNA-FA-Dab8γ, we treated S2-013 cells with Alexa Fluor 647-labeled chemically modified SNORA22 siRNA-FA-Dab8γ and SNORA22 siRNA-FA-Dab8α for 48 h. Confocal microscopy showed that the uptake of SNORA22 siRNA-FA-Dab8α by S2-013 cells was greater than that of SNORA22 siRNA-FA-Dab8γ (Fig. 3A), with a significantly higher number of cells incorporating SNORA22 siRNA-FA-Dab8α (Fig. 3B). Confocal microscopy confirmed FOLR1 expression on the cell membrane of PDAC cell lines (S2-013 and S2-CP8) and HPNE cells, with significantly higher levels being detected in S2-013 and S2-CP8 cells than in HPNE cells (Fig. 3C). This differential expression was consistent with the subsequent observation that both S2-013 and S2-CP8 cells exhibited the significantly greater cytoplasmic accumulation of chemically modified SNORA22 siRNA-FA-Dab8α than HPNE cells (Fig. 3C). This enhanced uptake was further confirmed by quantifying fluorescent intensity, which was significantly higher in both PDAC cell lines than in HPNE cells (Fig. 3D).

### SNORA22 siRNA-FA-Dab8 nucleic acid compounds taken up by PDAC cells via endocytosis.

**Fig. 4:**
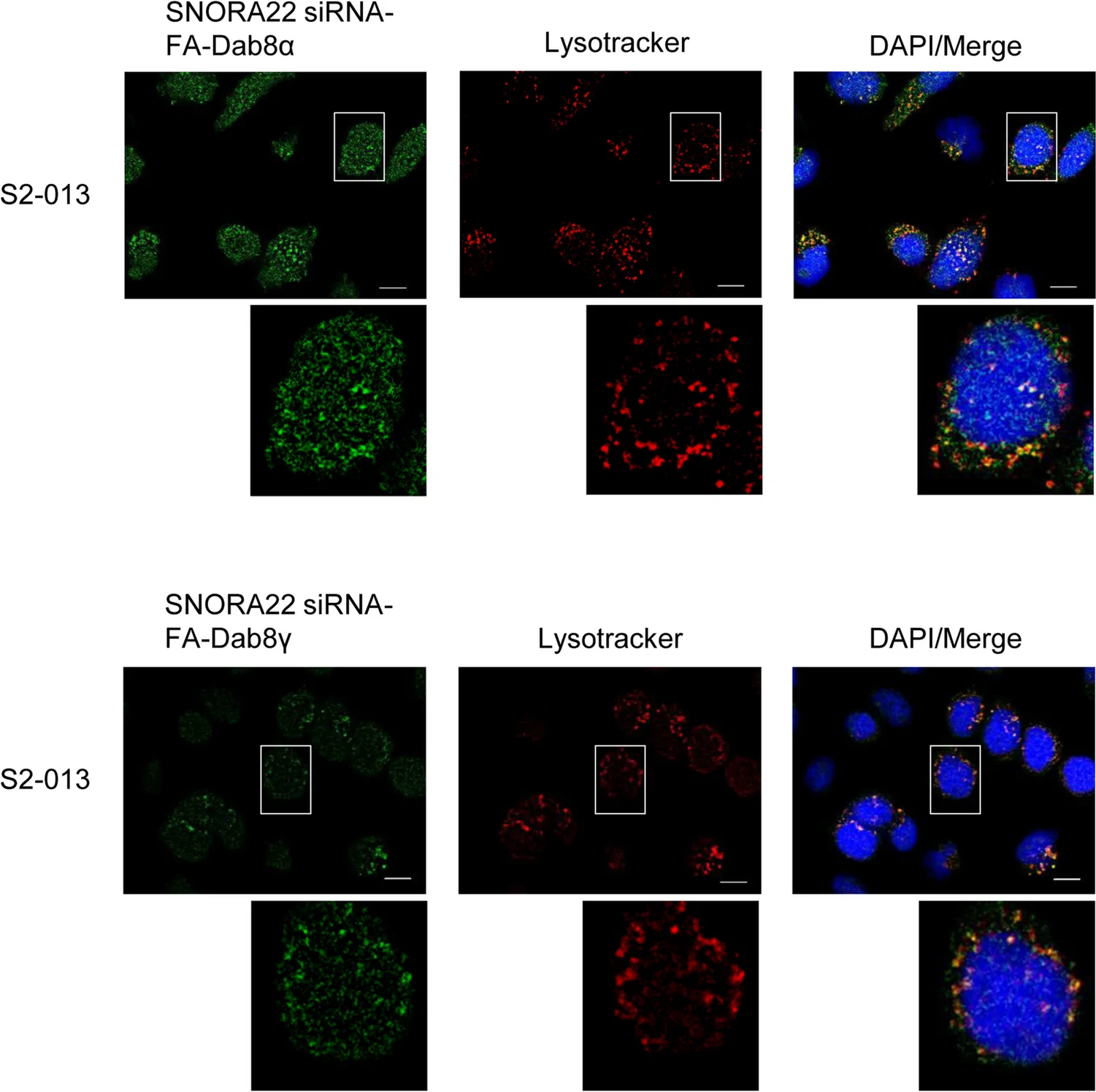
Localization of SNORA22 siRNA-FA-Dab8α and SNORA22 siRNA-FA-Dab8γ within lysosomes of S2-013 cells. Confocal immunofluorescence images show S2-013 cells incubated with Alexa Fluor 647-labeled chemically modified SNORA22 siRNA-FA-Dab8α and SNORA22 siRNA-FA-Dab8γ (green) and stained for lysosomes (LysoTracker, red) and nuclei (DAPI, blue). Cells enclosed in white boxes are shown at higher magnification below each panel to clearly demonstrate the specific colocalization of the nucleic acid compounds within the lysosomal compartments. Scale bars, 10 µm

To establish whether S2-013 cells internalized chemically modified SNORA22 siRNA-FA-Dab8α and SNORA22 siRNA-FA-Dab8γ via endocytosis, we added both Alexa Fluor 647-labeled compounds to cell culture media and incubated the cells for 48 h. Confocal microscopy revealed that, similar to the results shown in Fig. 3A, S2-013 cells took up more SNORA22 siRNA-FA-Dab8α than SNORA22 siRNA-FA-Dab8γ (Fig. 4). Furthermore, both compounds colocalized with Red DND-99-labeled lysosomes (labeled with LysoTracker) in S2-013 cells, suggesting endocytosis as the internalization mechanism (Fig. 4). This colocalization with lysosomes indicated that the compounds were successfully trafficked via the endocytic pathway, which is a necessary initial step for the release of the siRNA payload into the cytosol, where it then exerts its RNA interference effect. This DDS is specifically designed to facilitate this essential endosomal/lysosomal escape, consistent with established mechanisms for effective siRNA delivery.

### Expression profiles of *SNORA22* in public datasets

To evaluate the cancer selectivity and potential clinical relevance of our targeted therapeutic strategy across PDAC, we analyzed public transcriptomic datasets. Cross-tissue comparison using the RefEx database revealed that baseline *SNORA22* expression in normal human pancreatic tissue was below detectable limits (0 TPM), with expression restricted to a few specific tissues such as the cerebrum and adrenal gland (Supplementary Fig. 4A). In contrast, analysis of TCGA datasets demonstrated consistent *SNORA22* expression in human PDAC tissue specimens (Supplementary Fig. 4B). Combined with the marked overexpression of FOLR1 in PDAC cells relative to normal pancreatic epithelial cells (Fig. 3C, D) [20], these transcriptomic data indicate that normal pancreatic tissue inherently lacks both the delivery gateway (FOLR1) and the molecular target (*SNORA22*). Consistent with these expression profiles, normal pancreatic epithelial cells exhibited minimal siRNA uptake and no target knockdown.

### Roles of SNORA22 siRNA-FA-Dab8 nucleic acid compounds in invasion by PDAC cells.

To determine whether the suppression of tumor growth by *SNORA22* knockdown was due to direct anti-proliferative or pro-apoptotic effects, we performed MTT, Caspase 3/7, and Ki-67 assays in vitro. The complex of chemically modified SNORA22 siRNA and FA-Dab8α did not significantly alter cell viability (MTT assay) or apoptosis rates (Caspase 3/7 assay) compared to control groups (Supplementary Fig. 5A, B). Furthermore, confocal immunofluorescence microscopy revealed similar Ki-67 staining patterns across the control and experimental groups (Supplementary Fig. 5C). These findings demonstrate that *SNORA22* knockdown primarily inhibits lamellipodia-mediated invasion rather than directly affecting cell proliferation or apoptosis.

To confirm the downstream signaling effects of *SNORA22* knockdown, a phospho-protein array analysis was performed. Consistent with our proposed mechanism, the SNORA22 siRNA-FA-Dab8α complex markedly attenuated the phosphorylation of p70 S6 kinase (p70S6K), a crucial downstream effector of the mTORC1 pathway essential for lamellipodia formation (Supplementary Fig. 6A). Notably, the array also revealed a pronounced reduction in the phosphorylation levels of Src and RSK1/2, alongside a concurrent increase in the phosphorylation of MSK1/2 and Akt1/2/3 (Supplementary Fig. 6A). To further validate these key array findings, western blot analysis of p70S6K and Akt1/2/3 was performed (Supplementary Fig. 6B). Consistent with the array results, the chemically modified SNORA22 siRNA-FA-Dab8α complex noticeably decreased p70S6K phosphorylation while increasing Akt1/2/3 phosphorylation compared with the chemically modified SNORA22 siRNA–Dab8 complex, confirming the specific modulation of the mTORC1–Akt signaling axis.

**Fig. 5:**
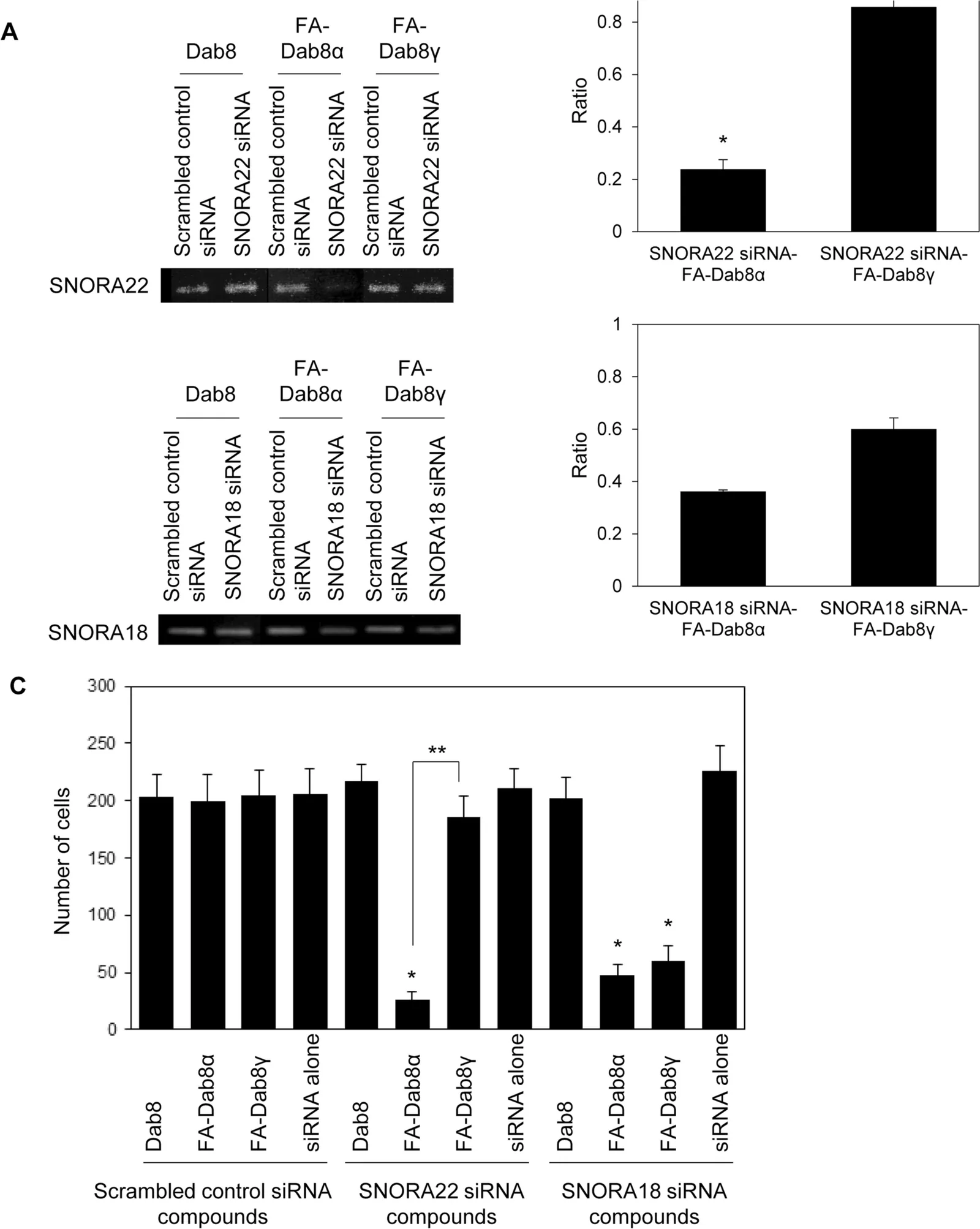
Roles of SNORA22 siRNA-FA-Dab8 nucleic acid compounds in invasion by PDAC cells. **A** Semi-quantitative RT-PCR analysis of SNORA22 following the incubation of chemically modified SNORA22 siRNA-FA-Dab8α and SNORA22 siRNA-FA-Dab8γ in the cultured media of S2-013 cells (upper panel). Similar experiments were performed with *SNORA18* (lower panel).**B** Quantification of data shown in A: *SNORA22* expression levels in S2-013 cells incubated with chemically modified SNORA22 siRNA-FA-Dab8α and SNORA22 siRNA-FA-Dab8γ. Data represent the mean ± SD derived from three independent experiments. SNORA22 levels, estimated by densitometry, were normalized to *SNORA22* levels in S2-013 cells incubated with scrambled control siRNA-FA-Dab8α and scrambled control siRNA-FA-Dab8γ, respectively. Results are presented as the ratio of SNORA22 in S2-013 cells incubated with chemically modified SNORA22 siRNA-FA-Dab8α and SNORA22 siRNA-FA-Dab8γ to SNORA22 in the corresponding S2-013 cells incubated with scrambled control siRNA-FA-Dab8α and scrambled control siRNA-FA-Dab8γ. Similar experiments were performed with *SNORA18*. *p < 0.05 significantly different from SNORA22 siRNA-FA-Dab8γ (the Student’s *t*-test).**C** Matrigel invasion assays were conducted using S2-013 cells treated with the following: scrambled control siRNAs (conjugated to Dab8, FA-Dab8α, or FA-Dab8γ), SNORA22 siRNAs (conjugated to Dab8, FA-Dab8α, or FA-Dab8γ), *SNORA18* siRNAs (conjugated to Dab8, FA-Dab8α, or FA-Dab8γ), and siRNA alone. Invading cells were counted in four randomly selected fields of view per group. Data are derived from three independent experiments. *Columns*, mean; *bars*, SD. *p <0.05 significantly different from scrambled control siRNA compounds. **p <0.05 significantly different from SNORA22 siRNA-FA-Dab8γ (the Student’s *t*-test)

Semi-quantitative RT-PCR showed that chemically modified SNORA22 siRNA-FA-Dab8α significantly decreased *SNORA22* expression in S2-013 cells, whereas chemically modified SNORA22 siRNA-FA-Dab8γ failed to suppress its expression (Fig. 5A). Furthermore, the extent of *SNORA22* suppression was confirmed by a quantitative analysis (Fig. 5B), which demonstrated that SNORA22 siRNA-FA-Dab8α induced a significantly stronger reduction than SNORA22 siRNA-FA-Dab8γ. In experiments targeting *SNORA18*, SNORA18 siRNA-FA-Dab8α and SNORA18 siRNA-FA-Dab8γ both achieved SNORA18 knockdown, with SNORA18 siRNA-FA-Dab8α exerting a stronger suppressive effect on *SNORA18* expression than SNORA18 siRNA-FA-Dab8γ (Fig. 5A, B).

Two-chamber invasion assays revealed that chemically modified SNORA22 siRNA-FA-Dab8α reduced the invasiveness of S2-013 cells significantly more than scrambled siRNA controls and SNORA22 siRNA-Dab8, whereas the invasion-suppressive effects of SNORA22 siRNA-FA-Dab8γ were similar to those of scrambled siRNA controls and SNORA22 siRNA-Dab8 (Fig. 5C). Consistent with the results shown in Fig. 5A, chemically modified SNORA22 siRNA-FA-Dab8α exerted a significantly stronger inhibitory effect on invasiveness than chemically modified SNORA22 siRNA-FA-Dab8γ.

In the *SNORA18* experiment, SNORA18 siRNA-FA-Dab8α inhibited cell invasion more strongly than SNORA18 siRNA-FA-Dab8γ (Fig. 5C). This result is consistent with the *SNORA18* suppression data presented in Fig. 5A. However, overall suppression achieved by *SNORA18* was significantly less potent than that by SNORA22 siRNA-FA-Dab8α (Fig. 5C). Based on the superior inhibition demonstrated by the *SNORA22* target, we selected *SNORA22* for subsequent drug efficacy evaluation experiments in a mouse model.

### Delivery of SNORA22 siRNA-FA-Dab8 nucleic acid compounds to xenografts in the S2-013-organoid model.

**Fig. 6:**
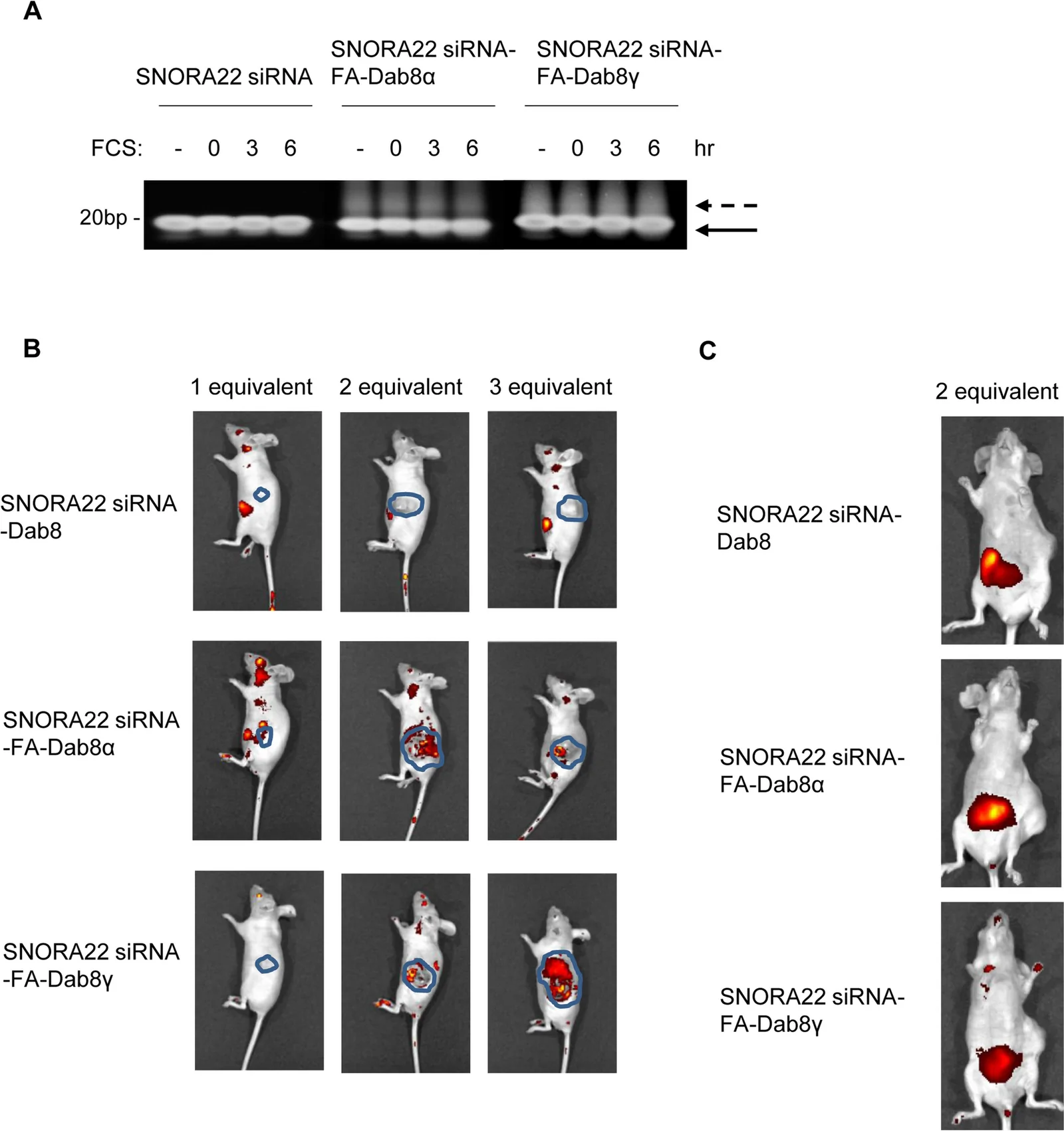
Delivery of SNORA22 siRNA-FA-Dab8 nucleic acid compounds to xenografts of the S2-013-organoid model. **A** Representative EMSA data using a polyacrylamide gel, showing the separation of chemically modified SNORA22 siRNA conjugated with FA-Dab8α and FA-Dab8γ, and free chemically modified SNORA22 siRNA, incubated with or without serum for 6 h. The dashed arrow represents chemically modified SNORA22 siRNA conjugated with FA-Dab8α and FA-Dab8γ. The solid arrow represents chemically modified SNORA22 siRNA without FA-Dab8α and FA-Dab8γ. A numerical size label (20 bp) is indicated on the left. **B** Seven weeks after implantation of the S2-013-derived organoid, Alexa Fluor 647-labeled, chemically modified SNORA22 siRNA-Dab8, SNORA22 siRNA-FA-Dab8α, and SNORA22 siRNA-FA-Dab8γ synthesized with 1, 2, and 3 equivalents were administered via a tail vein injection to the S2-013-organoid model (n=4). Twenty-four hours later, fluorescence images of Alexa Fluor 647-labeled SNORA22 siRNA in the xenografts were captured using an IVIS Spectrum In Vivo Imaging System. The blue-outlined region shows the xenograft. **C** Representative data showing the biodistribution of nucleic acid compounds synthesized at 2 equivalents in mice with no subcutaneous S2-013 cell line-derived organoid implantation. The compounds were primarily renally excreted. No fluorescence signal was detected in the liver

EMSA was used to analyze the interaction between chemically modified *SNORA22* siRNA and FA-Dab8α or FA-Dab8γ in serum. Although EMSA is known to potentially induce the dissociation of nucleic acid–protein complexes during electrophoresis [34], the binding of SNORA22 siRNA, retained in serum for 6 h, to FA-Dab8α and FA-Dab8γ did not significantly differ from that in serum-free controls (Fig. 6A).

To investigate whether chemically modified SNORA22 siRNA-FA-Dab8α and SNORA22 siRNA-FA-Dab8γ were delivered to PDAC tissues in vivo, a nude mouse model of PDAC was used. This model was established by subcutaneously implanting S2-013-derived organoids into the dorsal region of mice (S2-013-organoid model) [24]. Alexa Fluor 647-labeled chemically modified SNORA22 siRNA-Dab8, SNORA22 siRNA-FA-Dab8α, and SNORA22 siRNA-FA-Dab8γ were injected into nude mice via the tail vein 7 weeks after the subcutaneous implantation of human S2-013-derived organoids. Twenty-four hours after the injection of Alexa Fluor 647-labeled nucleic acid compounds, in vivo imaging studies were performed. The accumulation of the complex of SNORA-siRNA and 1, 2, or 3 equivalents of FA-Dab8α or FA-Dab8γ in xenografts of the S2-013-organoid model was examined. The complex of chemically modified SNORA22 siRNA and 2 equivalents of FA-Dab8α and that of chemically modified SNORA22 siRNA and 3 equivalents of FA-Dab8γ accumulated at higher levels in the xenografts than the other complexes (Fig. 6B). In S2-013-organoid mice without subcutaneous S2-013 cell line-derived organoid implantation, the nucleic acid compounds synthesized at 2 equivalents were renally excreted, and no fluorescence signal was observed in the liver (Fig. 6C).

### Inhibitory effects of SNORA22 siRNA-FA-Dab8 complexes on the tumor growth of xenografts in the S2-013-organoid model.

To examine the effects of SNORA22 siRNA-FA-Dab8α-based treatments on tumor growth inhibition in vivo, the S2-013-organoid model was used [24]. One week after the implantation of S2-013-derived organoids, mice in the following groups received their first treatment: scrambled control siRNA-FA-Dab8α, the complex of chemically modified SNORA22 siRNA and 2 equivalents of Dab8, the complex of chemically modified SNORA22 siRNA and 2 equivalents of FA-Dab8α, and GP. Since nab-paclitaxel is a combination of paclitaxel and human albumin, we used paclitaxel instead of nab-paclitaxel in this mouse experiment due to concerns about potential allergic reactions to human albumin in mice, as previously reported [35]. Treatment details are described in the Materials and Methods section.

**Fig. 7:**
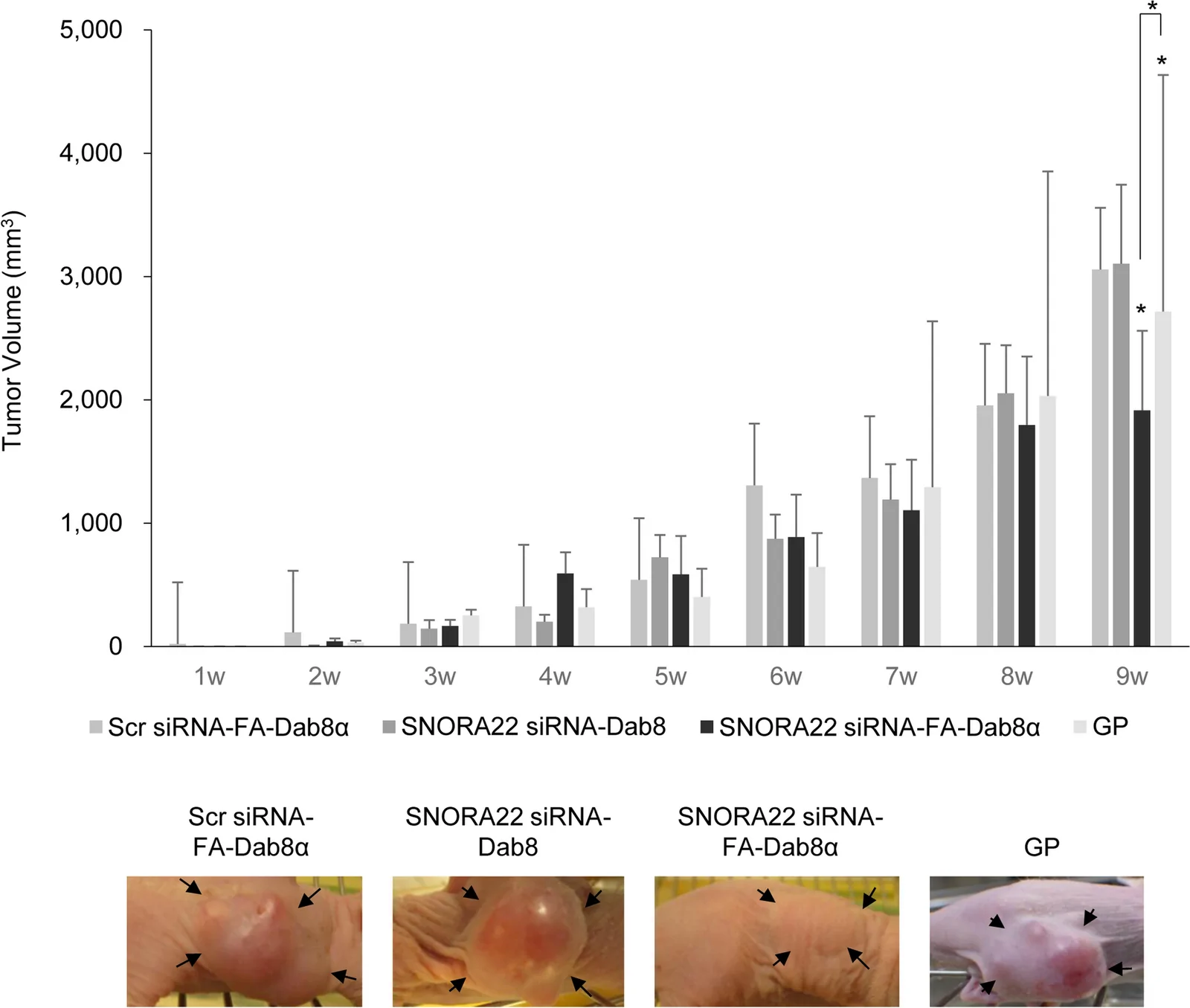
Inhibitory effects of a SNORA22 siRNA-FA-Dab8α nucleic acid compound on tumor growth in xenografts of the S2-013-organoid model. One week after the implantation of S2-013-derived organoids, mice in the scrambled control siRNA-FA-Dab8α (Scr-siRNA-FA-Dab8α), chemically modified SNORA22 siRNA-Dab8, and chemically modified SNORA22 siRNA-FA-Dab8α (n=8 each) groups began treatment, which continued for nine weeks. GP groups began treatment three weeks after implantation for six weeks. Average tumor volumes and representative images of mice in each treatment group were obtained every week. The images show the gross appearance of the mice. *Columns*, mean; *bar*s, SEM. Tumor volumes were compared weekly between the groups. *p <0.05 significantly different from Scr-siRNA-FA-Dab8α, SNORA22 siRNA-Dab8, and GP (the Student’s *t*-test). Arrows indicate xenografts (lower panels)

Tumor suppression after 9 weeks of treatment was significantly greater in the complex of chemically modified SNORA22 siRNA and 2 equivalents of FA-Dab8α group than in the other groups, including the GP group (Fig. 7). The efficacy of GP was also superior to that of scrambled control siRNA-FA-Dab8α and chemically modified SNORA22 siRNA-Dab8. Chemically modified SNORA22 siRNA-FA-Dab8α exhibited higher efficacy than GP. Furthermore, chemically modified SNORA22 siRNA-Dab8 suppressed tumor growth only in the presence of FA. In the absence of FA, no significant difference was observed between the chemically modified SNORA22 siRNA-Dab8 group and scrambled control siRNA-FA-Dab8α group.

### Histopathological evaluation after the treatment with a SNORA22 siRNA-FA-Dab8α nucleic acid compound in xenografts of the S2-013-organoid model.

**Fig. 8:**
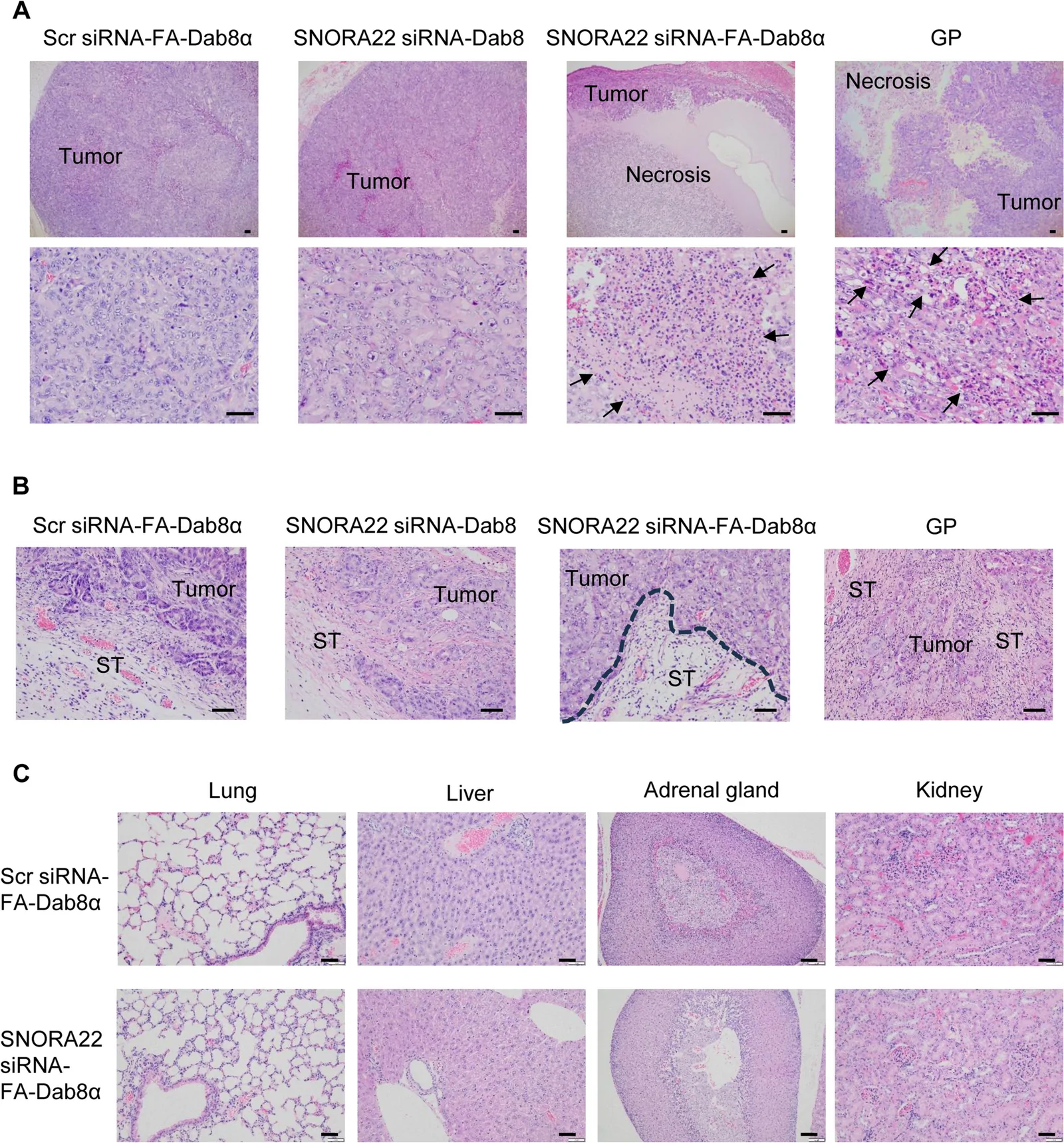
Histopathological evaluation after treatment with a SNORA22 siRNA-FA-Dab8α nucleic acid compound in xenografts of the S2-013-organoid model. **A, B** Representative H&E staining of tumor xenografts obtained from mice nine (GP group) or ten (all other groups) weeks after transplantation. Tumor necrosis (**A**) and peritumoral subcutaneous infiltration (**B**) are shown for the scrambled control siRNA-FA-Dab8α (Scr-siRNA-FA-Dab8α)-treated control group, the SNORA22 siRNA-Dab8-treated group, the SNORA22 siRNA-FA-Dab8α-treated group, and the GP-treated group. Arrows, tumor necrosis; dotted lines, the tumor-infiltrating margin; ST, subcutaneous tissue. Scale bars, 50 µm. **C** Representative H&E-stained tissue sections from lungs, liver, adrenal glands, and kidneys of mice following the administration of scrambled control siRNA-FA-Dab8α or chemically modified SNORA22 siRNA-FA-Dab8α

Nine (GP group) or ten (all groups other than the GP group) weeks after implantation, mice were sacrificed. Xenograft sections were prepared and stained with H&E to evaluate potential histopathological changes in the tissue architecture caused by the SNORA22 siRNA-FA-Dab8α-based treatments (Fig. 8A, B). The necrotic area within the tumor was markedly larger after the treatment with the complex of chemically modified SNORA22 siRNA and 2 equivalents of FA-Dab8α than after the treatment with chemically modified SNORA22 siRNA-Dab8 (Fig. 8A). The treatment with SNORA22 siRNA-FA-Dab8α clearly increased the area of tumor necrosis. Additionally, a significant difference was observed in the extent of necrotic areas within the xenografts between the chemically modified SNORA22 siRNA-FA-Dab8α group and GP group (Fig. 8A); the chemically modified SNORA22 siRNA-FA-Dab8α group exhibited a larger necrotic area within the xenografts than the GP group.

Adenocarcinoma with the regional invasion of subcutaneous tissue was observed in xenografts from all treatment groups (Fig. 8B). Similar infiltrative findings were noted in xenografts after the treatments with scrambled siRNA control-FA-Dab8α, chemically modified SNORA22 siRNA-Dab8, and GP (Fig. 8B). In contrast, chemically modified SNORA22 siRNA-FA-Dab8α reduced subcutaneous tissue infiltration more than the other groups (Fig. 8B). Therefore, the complex of chemically modified SNORA22 siRNA and 2 equivalents of FA-Dab8α appeared to suppress tumor growth via two mechanisms: the induction of necrosis in tumor cells and the inhibition of invasion into surrounding subcutaneous tissues.

To further evaluate the overall invasive growth pattern across the entire tumor architecture, macroscopic photographs of the resected tumors and low-magnification H&E-stained images of whole tumor sections were examined (Supplementary Fig. 7). These evaluations demonstrated that tumors treated with the SNORA22 siRNA-FA-Dab8α complex exhibited clear, well-demarcated boundaries with surrounding normal tissues, in stark contrast to the highly infiltrative tumor margins and regional tissue invasion observed in the control and GP-treated groups.

Following the administration of scrambled siRNA control-FA-Dab8α and chemically modified SNORA22 siRNA-FA-Dab8α, a comprehensive histopathological examination of the major organs was performed (Fig. 8C). This detailed microscopic analysis of tissue sections—specifically from the lungs, liver, adrenal glands, and kidneys—revealed no discernible pathological lesions or structural abnormalities. Additionally, histopathological evaluations of the heart and spleen also showed no abnormal findings (data not shown). These results indicate that the compound was tolerated well and did not induce any detectable tissue damage or toxicity at the cellular or architectural level in these vital organs.

## Discussion

The mortality rate from PDAC continues to increase not only in Japan, but also in Western countries. In 2023, approximately 44,000 individuals were diagnosed with PDAC, and nearly 40,000 succumbed to the disease, ranking it as the fourth leading cause of cancer-related deaths in Japan [36]. With a 5 year survival rate of only 5–10%, and the annual number of deaths closely mirroring the number of diagnoses, PDAC remains one of the most lethal malignant tumors. Limited therapeutic options highlight the urgent need for new and effective treatments.

The peritumoral environment of PDAC is notoriously challenging for drug delivery, making it difficult to translate drug efficacy from in vitro to in vivo studies. A recent review by Viegas et al. on nano-based drug delivery systems for PDAC underscores these difficulties, noting the dense fibrotic stroma and high interstitial pressure as key obstacles [37]. This is the first study to successfully demonstrate the anticancer efficacy of a nucleic acid drug using a novel DDS specifically targeting PDAC, with positive results in both in vitro and in vivo settings. The ability of this new DDS to overcome the significant hurdles of drug delivery to PDAC suggests the potential of this groundbreaking approach for other types of cancer in the future.

While clinical trials are examining molecularly targeted therapies and immune checkpoint inhibitors for PDAC, current treatment efficacy remains limited. A cancer genome analysis of PDAC commonly reveals mutations in four key genes: KRAS, CDKN2A/p16, TP53, and SMAD4/DPC4 [38]. Although clinical trials are evaluating KRAS G12C inhibitors and pan-RAF inhibitors for unresectable lung cancer, drugs targeting the three other gene abnormalities are still being developed. PARP inhibitors, such as olaparib, have demonstrated efficacy for PDAC with BRCA gene mutations, similar to their use for hereditary breast and ovarian cancer syndrome [39]. Olaparib is approved as maintenance therapy following mFFX for BRCA mutation-positive, unresectable PDAC. However, only approximately 7% of PDAC cases harbor BRCA mutations, and its restriction to maintenance therapy limits its applicability.

Our research aims to elucidate the mechanisms driving the invasiveness and metastasis of PDAC cells. We initially observed the formation of multiple lamellipodia in invading cells, prompting our investigation of these structures [31]. This led to the identification of key signaling pathways and molecules involved in PDAC cell invasiveness [40]. To target these molecules, we designed siRNAs and developed a delivery system using nanoparticles composed of cationic polymers (PEG and COL) and FA, the receptor for which is highly expressed on PDAC cell membranes [20]. In a conventional PDAC xenograft model (S2-013 cells transplanted into the mouse pancreas), the intravenous administration of siRNAs targeting ARHGEF4, CCDC88A, LAMTOR2, the mechanistic target of rapamycin (mTOR), NUP85, and WASF2 suppressed direct peritoneal invasion and improved prognosis [20]. Further studies revealed that snoRNAs, including *SNORA22*, which accumulates in lamellipodia, play a crucial role in their formation and contribute significantly to PDAC cell invasion [23]. In the same xenograft model, siRNA targeting *SNORA22* suppressed both direct peritoneal invasion and distant metastasis, resulting in an improved prognosis [23].

The research and development of nucleic acid drugs have experienced significant growth, driven by recent approvals for intractable diseases such as hereditary amyloid transthyretin amyloidosis [41]. The first siRNA drug, patisiran, was approved by the U.S. Food and Drug Administration for the treatment of acute hepatic porphyria in adults [42]. Its long-term safety and efficacy have been confirmed in extension studies. At least six siRNA drugs have now been approved in the U.S., Europe, and Japan [43]. Patisiran is a nucleic acid drug that knocks down TTR mRNA, which utilizes lipid nanoparticles with the ApoE protein as a DDS [44]. However, the field is diverse; of the seven approved siRNA drugs, six—including givosiran—employ a different strategy: a ligand-conjugated DDS, specifically a GalNAc ligand. This success highlights the crucial importance of an effective DDS, particularly active targeting, to ensure that drugs reach the correct tissues. In cancer drug discovery, nucleic acid drugs are highly anticipated as the next generation of therapeutics, following antibody drugs. Despite their significant market potential for malignancies, including PDAC, few have advanced to clinical trials globally. Current challenges involve identifying relevant RNA targets and, most importantly, developing an effective DDS to deliver these drugs to the affected tissues. The success of active targeting in other approved nucleic acid drugs suggests that it will be a critical factor in developing effective treatments for PDAC.

To facilitate clinical translation, we are developing a chemically modified oligonucleotide agent targeting *SNORA22* that is suitable for clinical use. We previously reported a DDS that reduced glomerular filtration-mediated excretion by augmenting the molecular weight and modulating the charge through PEGylation [20]. Building on this, we have generated a lead compound targeting *SNORA22*, utilizing a modified DDS with FA-Dab8 instead of cationic polymer nanoparticles for improved delivery. We selected Dab8 for this DDS due to its eight amino groups, which are optimally located for siRNA binding [21]. To enhance the targeting of PDAC cells, which highly express folate receptors, we conjugated FA to Dab8. To overcome the barrier of the hydrophobic lipid bilayer, the hydrophilic oligonucleotide is complexed with FA-Dab8, facilitating its efficient uptake through binding to folate receptors on the PDAC cell membrane. This targeted delivery enables the compound to access the cytoplasm and interact with SNORA22. This FA-Dab8 DDS is capable of stabilizing SNORA22 siRNA and delivering it specifically to PDAC tissue. Furthermore, to improve RNase resistance and bloodstream stability, the lead compound was optimized through phosphorothioate and 2’-O-Me modifications to pyrimidine bases. This chemically modified SNORA22 siRNA was then conjugated to FA-Dab8 DDS.

Although both SNORA22 siRNA-FA-Dab8α and SNORA22 siRNA-FA-Dab8γ were successfully trafficked to the lysosome, the α-regioisomer (FA-Dab8α) exhibited significantly higher cellular uptake and stronger functional knockdown potency in PDAC cells. Similarly, in experiments targeting *SNORA18*, the α-modified compound demonstrated superior functional potency, achieving stronger knockdown of *SNORA18* and inhibiting cell invasion more effectively than its γ-modified counterpart. These findings are particularly intriguing given the conflicting historical data, where some studies reported that conjugation to the γ-carboxy group was effective for efficient tumor uptake while the α-carboxy group was not [45, 46], whereas others concluded that α- and γ-conjugates exhibited similar binding affinities and delivery efficiencies [47, 48]. Based on the crystal structure of the human FOLR1–folic acid complex, the glutamate moiety of folic acid is exposed to the solvent, which theoretically allows for chemical modifications at either the α- or γ-carboxylate without disrupting core receptor recognition [49]. Therefore, the distinct phenotypic superiority of the α-conjugate observed in our study likely stems from macro-structural differences in the resulting polyplexes, rather than the intrinsic receptor-binding affinity of the ligand monomer itself. While detailed biophysical characterization of these polyplexes—such as particle size distribution and surface charge—remains an important subject for future physicochemical studies, our findings emphasize that regioisomer selection is a critical parameter when optimizing peptide-based delivery systems.

As demonstrated by our public database analyses (TCGA and RefEx) and in vitro assessments across multiple PDAC cell lines (S2-013 and S2-CP8), our FA-Dab8α delivery system achieves a robust dual-layer tumor selectivity [18–20, 23]. First, active targeted delivery is restricted by the cell-surface expression of FOLR1, which is consistently overexpressed in human PDAC tissues and cell lines[18, 19] [20, 50]but virtually absent in normal pancreatic epithelial cells (Fig. 3C, D; Supplementary Fig. 4) [20]. This confirms that FOLR1 serves as a widely applicable “delivery gateway” for PDAC-targeted therapy. Second, expression of the oncogenic snoRNA target, *SNORA22*, is specifically upregulated in PDAC tissues [23] while remaining undetectable in normal pancreatic tissues (0 TPM in RefEx). Consequently, non-specific knockdown or toxicity in normal pancreatic cells is intrinsically prevented due to the absence of both target payload uptake and endogenous *SNORA22* expression. Histopathological examination of vital organs—including the lungs, liver, kidneys, and adrenal glands—revealed no observable structural damage or toxicity following systemic administration in the S2-013-organoid model. Notably, despite the compound being primarily cleared via renal excretion, the kidneys exhibited no histological abnormalities. These findings confirm the exceptional safety and high therapeutic potential of the FA-Dab8α delivery platform in vivo, fully supported by the dual-layer selectivity provided by FOLR1 expression and *SNORA22* target availability.

It is well established that the therapeutic efficacy of siRNA carrier platforms depends heavily on their nano-structural characteristics, including charge distribution, ligand density, and molecular packing, which govern cellular uptake and endosomal escape [17]. Notably, our in vivo imaging data revealed that the optimal complexation ratio for tumor accumulation differed between the two regioisomers, requiring 2 equivalents for FA-Dab8α and 3 equivalents for FA-Dab8γ. This variation strongly suggests that conjugation at the α-position of the glutamate moiety alters the packing efficiency or the spatial rearrangement of the Dab8–siRNA complex. Such structural alterations could spontaneously optimize the spatial orientation and surface density of the folic acid ligands on the nanostructure, thereby facilitating receptor-mediated endocytosis and subsequent endosomal/lysosomal escape. While a detailed structural analysis of these polyplexes remains an objective for future studies, our findings underscore the critical impact of regioisomer selection in the optimization of oligonucleotide delivery systems.

To establish whether our chemically modified oligonucleotide agent targeting *SNORA22* suppressed both tumor growth and infiltration from PDAC tissue into surrounding tissues, we conducted in vivo experiments. In oncology, many drug candidates fail to achieve proof of concept (POC), hindering their progression to clinical trials. Therefore, preclinical studies using relevant models are essential for establishing POC. Since conventional PDAC xenograft models often lack predictive power for clinical efficacy, a mouse model that closely mimics clinical PDAC is necessary. In PDAC, the tumor microenvironment, including the cancer stroma, plays a pivotal role in disease progression. PDAC cell infiltration into surrounding tissues is often dependent on stromal interactions, highlighting the need to assess drug efficacy against infiltration in models that incorporate this key component. The S2-013-organoid model meets these criteria, making it a promising tool for predicting drug efficacy.

We recently demonstrated in the S2-013-organoid model that rapamycin, a selective inhibitor of mTOR protein kinase, exerted its tumor growth inhibitory effect not by inhibiting cell proliferation, but primarily by suppressing tumor invasion [35]. This important finding strongly emphasizes that mTOR signaling plays a central role in driving the invasion of PDAC. In the present study, the chemically modified SNORA22 siRNA-FA-Dab8α complex was administered intravenously via a tail vein injection to the S2-013-organoid model. Fluorescence imaging 24 h post-administration confirmed the accumulation of the agent within the PDAC tissue of the xenografts. The agent significantly suppressed both tumor growth and invasiveness, suggesting its ability to inhibit the spread of PDAC from the primary tumor to surrounding tissues and potentially representing the first nucleic acid drug with this efficacy.

To elucidate the specific signaling cascades impacted by *SNORA22* suppression, we performed a phospho-protein array analysis. Crucially, the assay detected that the nucleic acid drug targeting *SNORA22* suppressed downstream effectors of mTOR signaling, mirroring the invasion-inhibitory phenotype previously observed with rapamycin [35]. Treatment with the SNORA22 siRNA-FA-Dab8α complex attenuated the phosphorylation of multiple downstream effectors and mediators in the mTOR signaling network. Notably, there was a pronounced reduction in the phosphorylation levels of p70S6K, Src and RSK1/2, and an increase in the phosphorylation of MSK1/2 and Akt1/2/3. The concurrent elevation of Akt phosphorylation following the suppression of p70S6K is a well-documented biochemical phenomenon caused by the release of the negative feedback loop mediated by insulin receptor substrate-1 (IRS-1) [51]. Furthermore, Carracedo et al. demonstrated that mTORC1 inhibition simultaneously leads to the compensatory activation of the MAPK pathway through a similar PI3K-dependent feedback loop [52]. Given that MSK1/2 are direct downstream effectors of the MAPK cascade, the increase in MSK1/2 phosphorylation observed in our study perfectly aligns with this established feedback mechanism. While the precise biochemical mediators connecting non-coding *SNORA22* to the mTOR/p70S6K kinase cascade remain to be fully mapped in future studies, these data demonstrate that targeting *SNORA22* effectively disrupts multiple concurrent pro-invasive signaling pathways, ultimately resulting in the suppression of lamellipodia-mediated invasion [23, 35].

A limitation of the current study is the reliance on a single siRNA sequence targeting *SNORA22*, which inherently carries a potential risk of sequence-dependent off-target effects within target cells. While our FOLR1-targeted FA-Dab8α delivery system effectively minimizes non-specific payload accumulation and systemic toxicity in normal tissues natively lacking both FOLR1 and *SNORA22*, validating intracellular sequence specificity remains important. Therefore, future preclinical investigations will comprehensively validate these phenotypic findings using multiple independent siRNA sequences targeting different regions of SNORA22, combined with rigorous in silico homology profiling, to definitively rule out off-target interference prior to clinical translation.

In conclusion, the S2-013-organoid model demonstrates significantly improved treatment efficacy with the chemically modified oligonucleotide agent targeting *SNORA22*, indicating the potential for human therapy with this single agent. Prior to non-clinical safety studies in animals, we will conduct in silico analyses to predict potential off-target toxicity caused by siRNA hybridization to unintended targets because the siRNA sequence targeting *SNORA22* is the active pharmaceutical ingredient. We will also perform expression analyses of *SNORA22* in various animal species (mouse, rat, dog, cynomolgus monkey, and pig) to select the appropriate species for non-clinical studies. These results will inform the final non-clinical study plan for investigating the safety of chemically modified oligonucleotide agents targeting *SNORA22*.

## Supplementary Information

Below is the link to the electronic supplementary material.

Supplementary file1 (PDF 904 KB)

## Data Availability

The data that support the findings of this study are available from the corresponding authors, Keisuke Taniuchi and Takeshi Wada, upon reasonable request.

## Abbreviations

*2′-O*-methyl,COL: Chitosan oligosaccharide lactate
Dab8: L-2,4-diaminobutyric acid octamer
DCC: *N,N’*-Dicyclohexylcarbodiimide
DDS: Drug delivery system
DMF: *N,N*-Dimethylformamide
EMSA: Electrophoretic mobility shift assay
FA: Folic acid
Fmoc: 9-Fluorenylmethyloxycarbonyl
FOLR1: Folate receptor alpha
GP: Gemcitabine plus paclitaxel
HUVEC: Human umbilical vein endothelial cell
IVIS: In vivo imaging system
KHSRP: KH-type splicing regulatory protein
MSC: Mesenchymal stem cell
mTOR: Mechanistic target of rapamycin
PDAC: Pancreatic ductal adenocarcinoma
PEG: Polyethylene glycol
PS: Phosphorothioate
RP-HPLC: Reverse-phase high-performance liquid chromatography
siRNA: Small interfering RNA
snoRNA: Small nucleolar RNA
SPPS: Solid-phase peptide synthesis
