# Supplementary material for: Inhibition of pancreatic cancer growth by an siRNA targeting SNORA22 and a folic acid-conjugated cationic oligopeptide
Source: Hum Cell. 2026 Sep 30;39(10):146. doi: 10.1007/s13577-026-01458-1 (PMC13627270; doi:10.1007/s13577-026-01458-1)
Supplement: Supplementary file 1 [file 13577_2026_1458_MOESM1_ESM.pdf]

Supplementary Figure 1

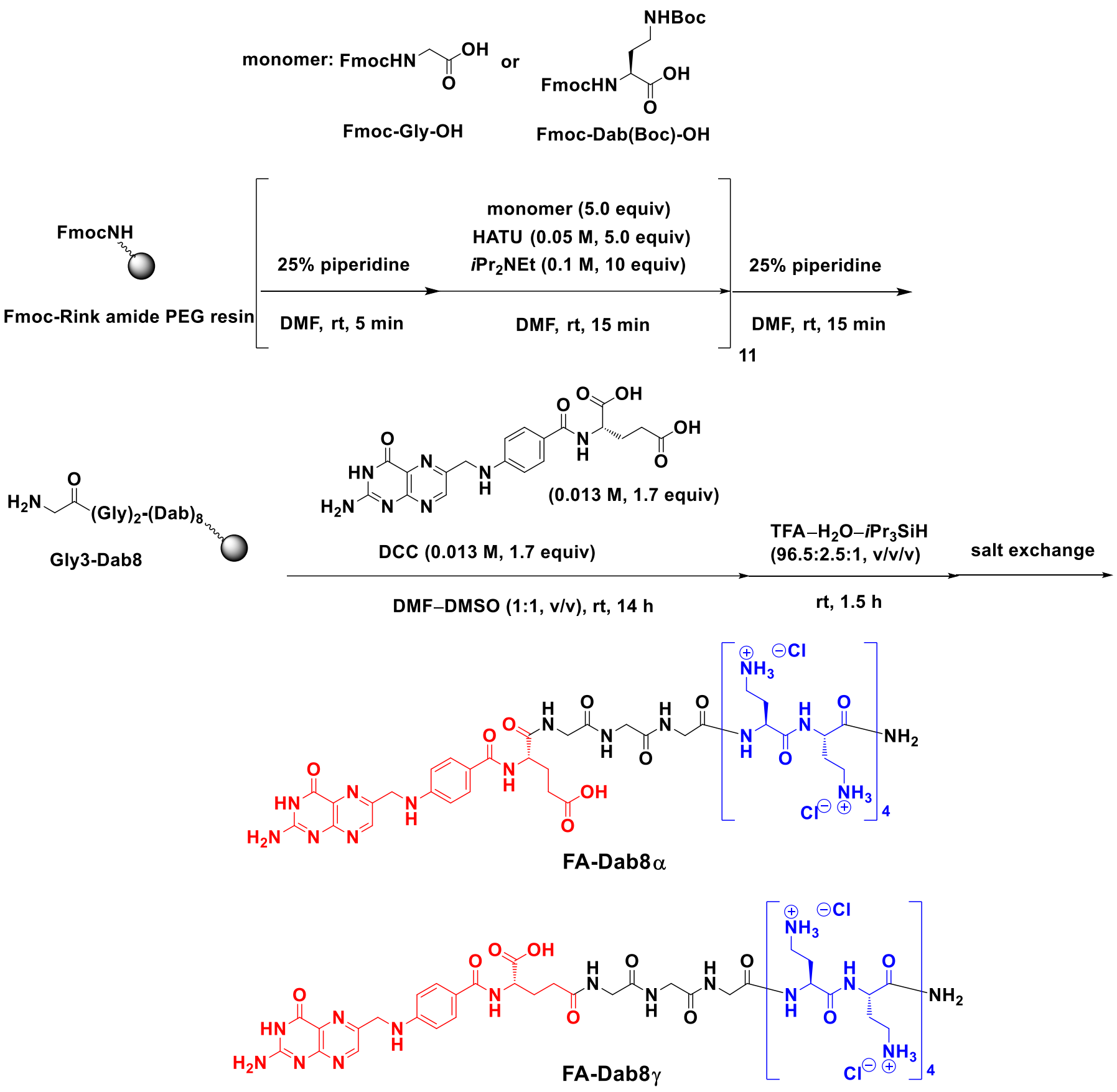

Supplementary Figure 1. Synthesis of FA-Dab8α and FA-Dab8γ.

Schematic of FA-Dab8 regioisomer synthesis via Fmoc SPPS on Rink amide-PEG resin. Folic acid was conjugated to the Gly3-Dab8 N terminus using DCC in DMF–DMSO. Global deprotection and resin cleavage yielded a crude mixture of regioisomers, which were subsequently purified by RP-HPLC.

Supplementary Figure 2

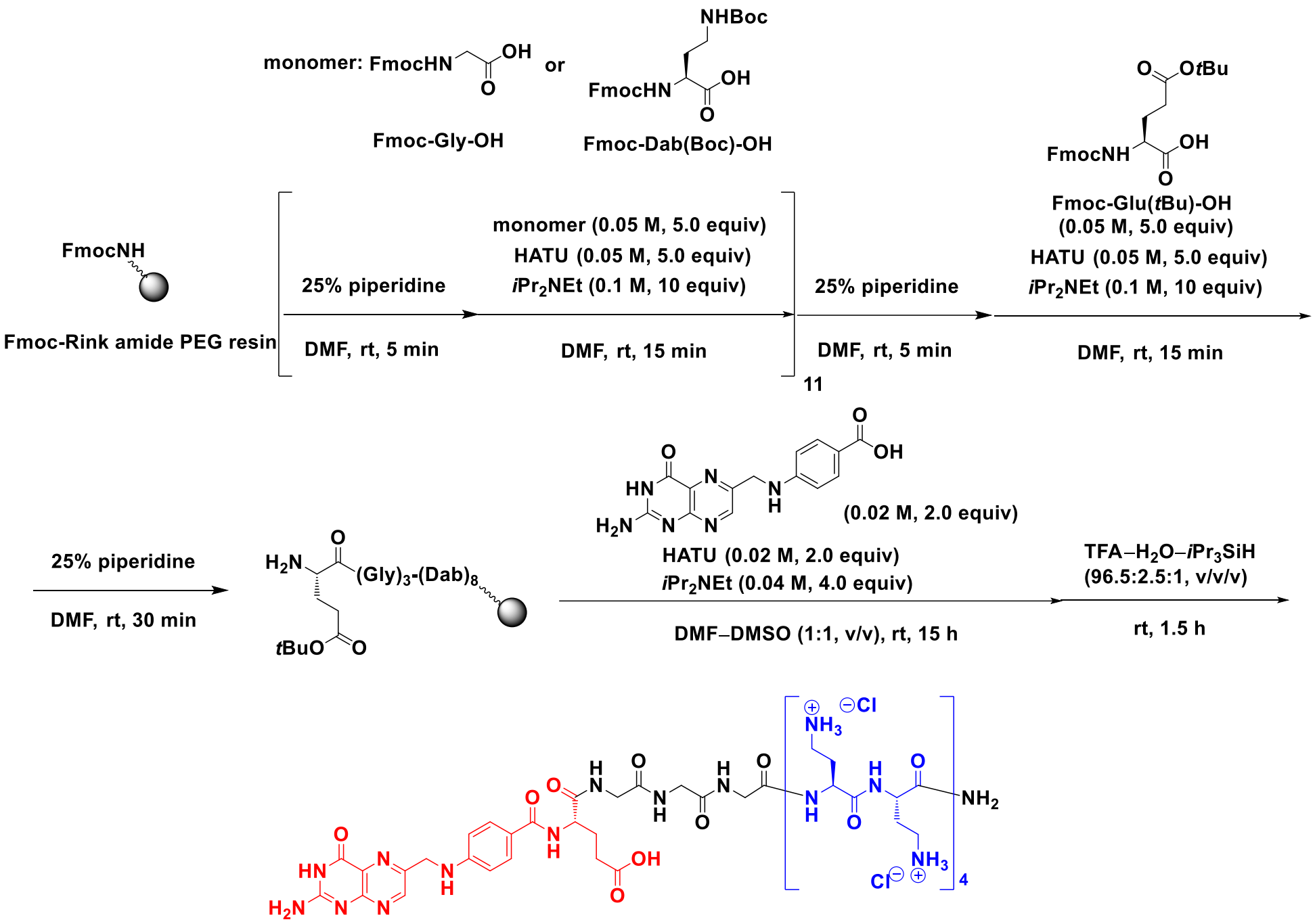

Supplementary Figure 2. Regioselective synthesis of FA-Dab8α.

Synthetic route for the α-regioisomer. After Fmoc-Glu(tBu)-OH coupling and Fmoc removal, pteroic acid was conjugated to the glutamate α-amino group. Global deprotection and cleavage afforded the pure FA-Dab8α.

Supplementary Figure 3

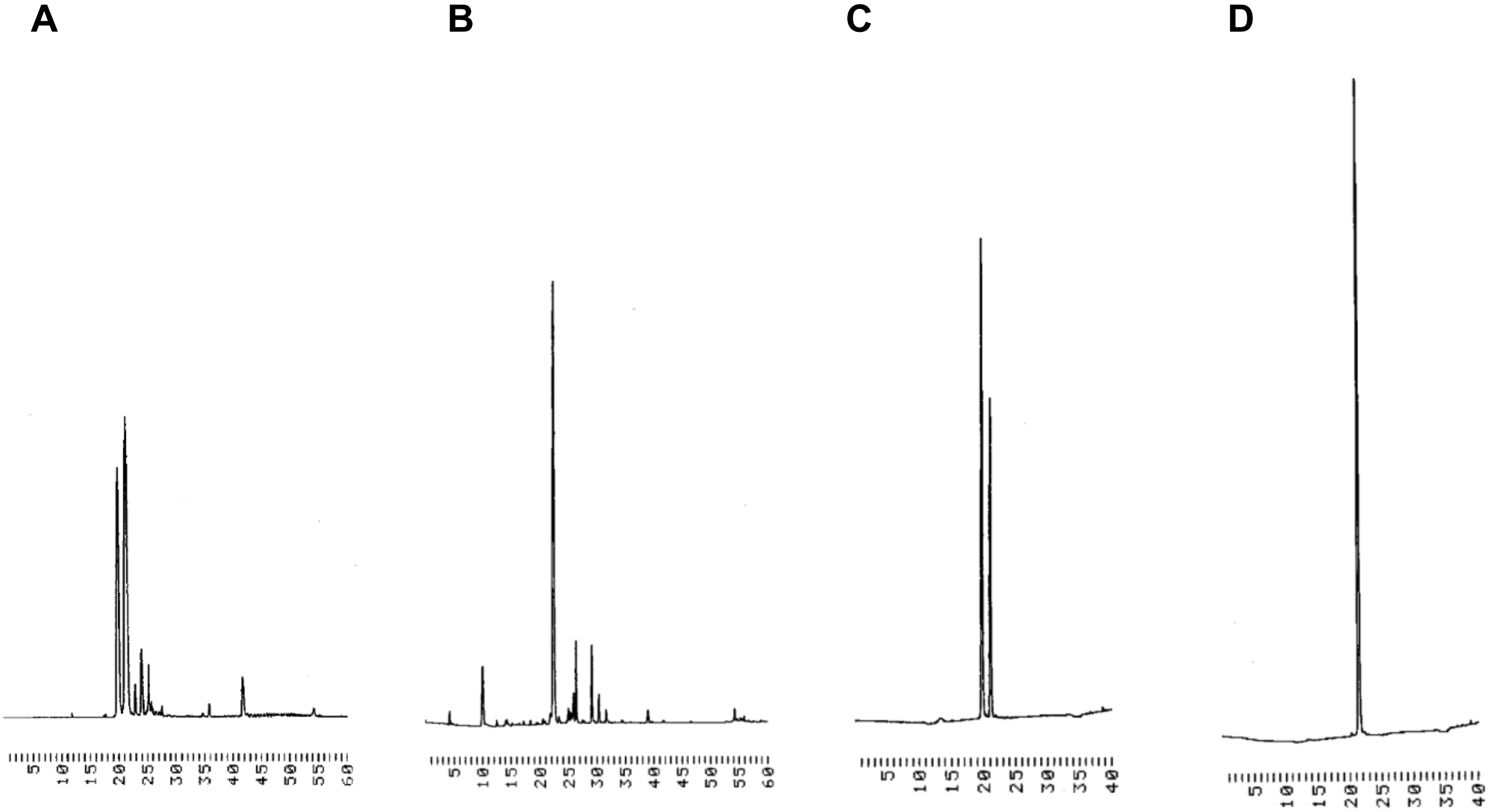

Supplementary Figure 3. RP-HPLC analysis and identification of FA-Dab8 regioisomers.

(A) RP-HPLC chromatogram of crude FA-Dab8 showing two peaks: FA-Dab8A (19 min) and FA-Dab8B (21 min).

(B) RP-HPLC of regioselectively synthesized FA-Dab8α.

(C, D) Co-injection of FA-Dab8α with FA-Dab8A (C) and FA-Dab8B (D). FA-Dab8A and FA-Dab8B were identified as FA-Dab8γ and FA-Dab8α, respectively.

Supplementary Figure 4

A

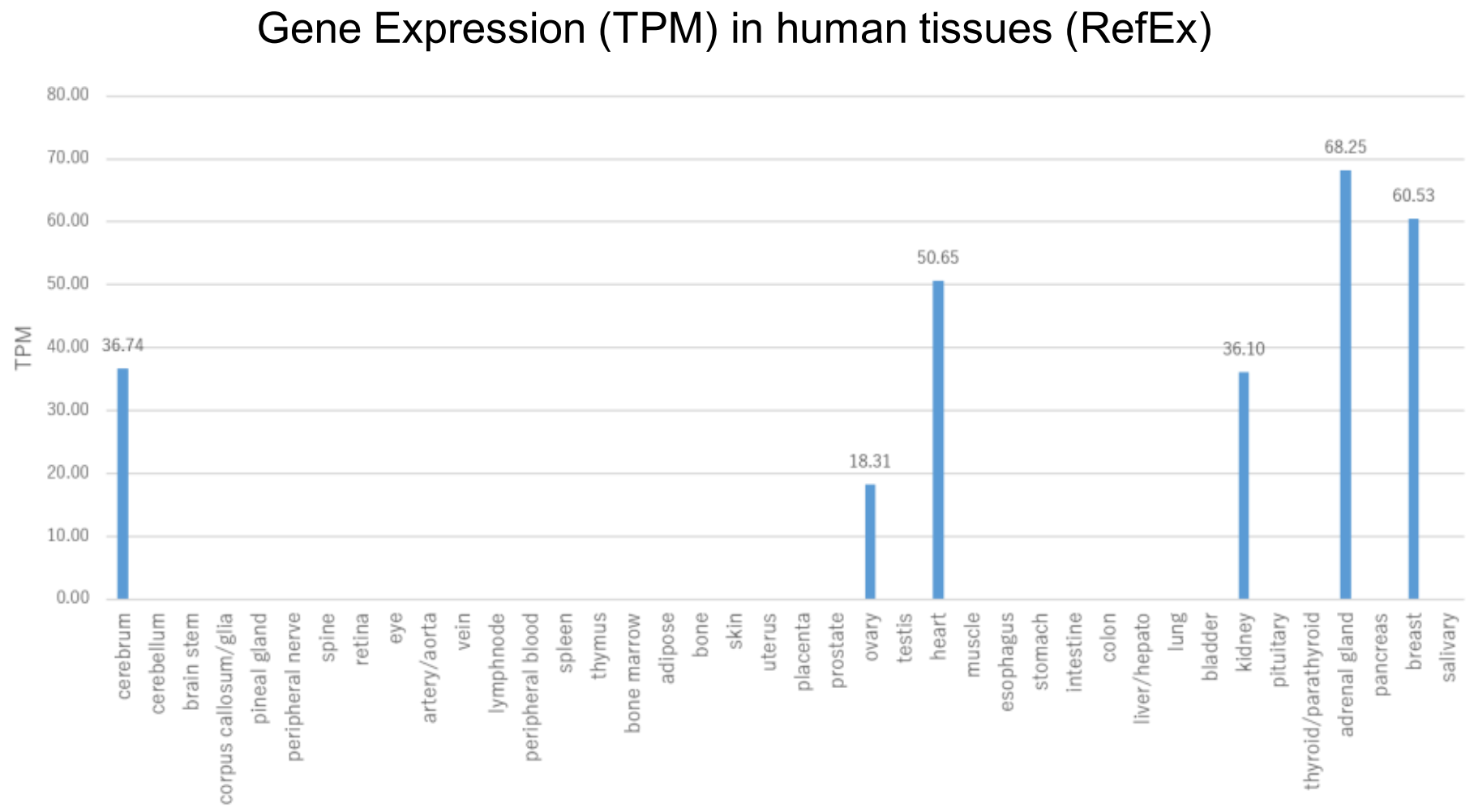

B

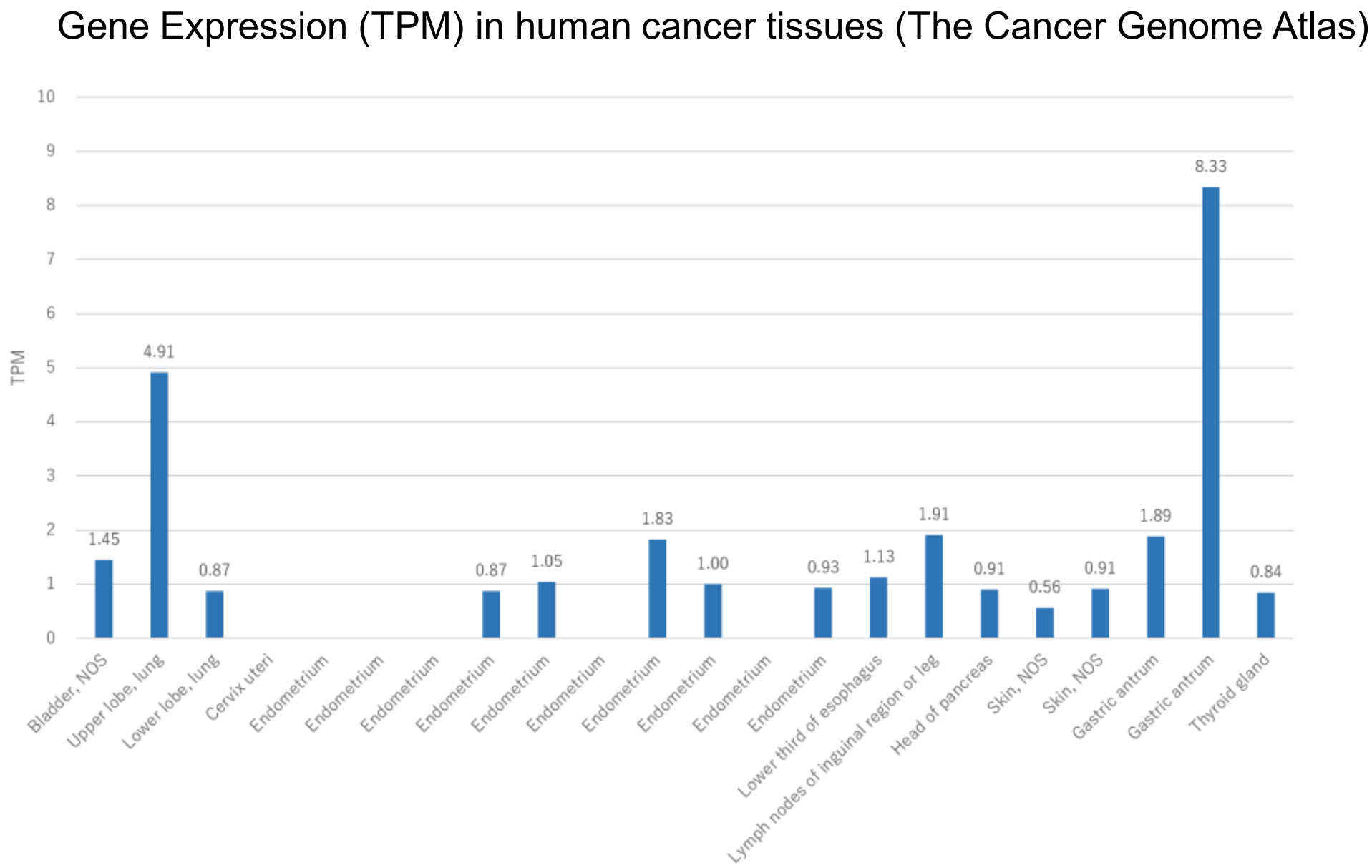

Supplementary Figure 4. Expression profiles of *SNORA22* in normal human tissues and various human cancer specimens from public datasets.

(A) Baseline expression levels of *SNORA22* across various normal human tissues retrieved from the Reference Expression dataset (RefEx). Expression values on the vertical axis are presented as Transcripts Per Million (TPM). Note that the expression level of *SNORA22* in normal human pancreatic tissue is below detectable limits (0 TPM).

(B) Transcriptome expression profiles of *SNORA22* across various human cancer specimens (including pancreatic head tumors) obtained from The Cancer Genome Atlas (TCGA) database. Expression values on the vertical axis are presented as TPM, demonstrating consistent upregulation and strong expression of *SNORA22* in PDAC tissues.

Supplementary Figure 5

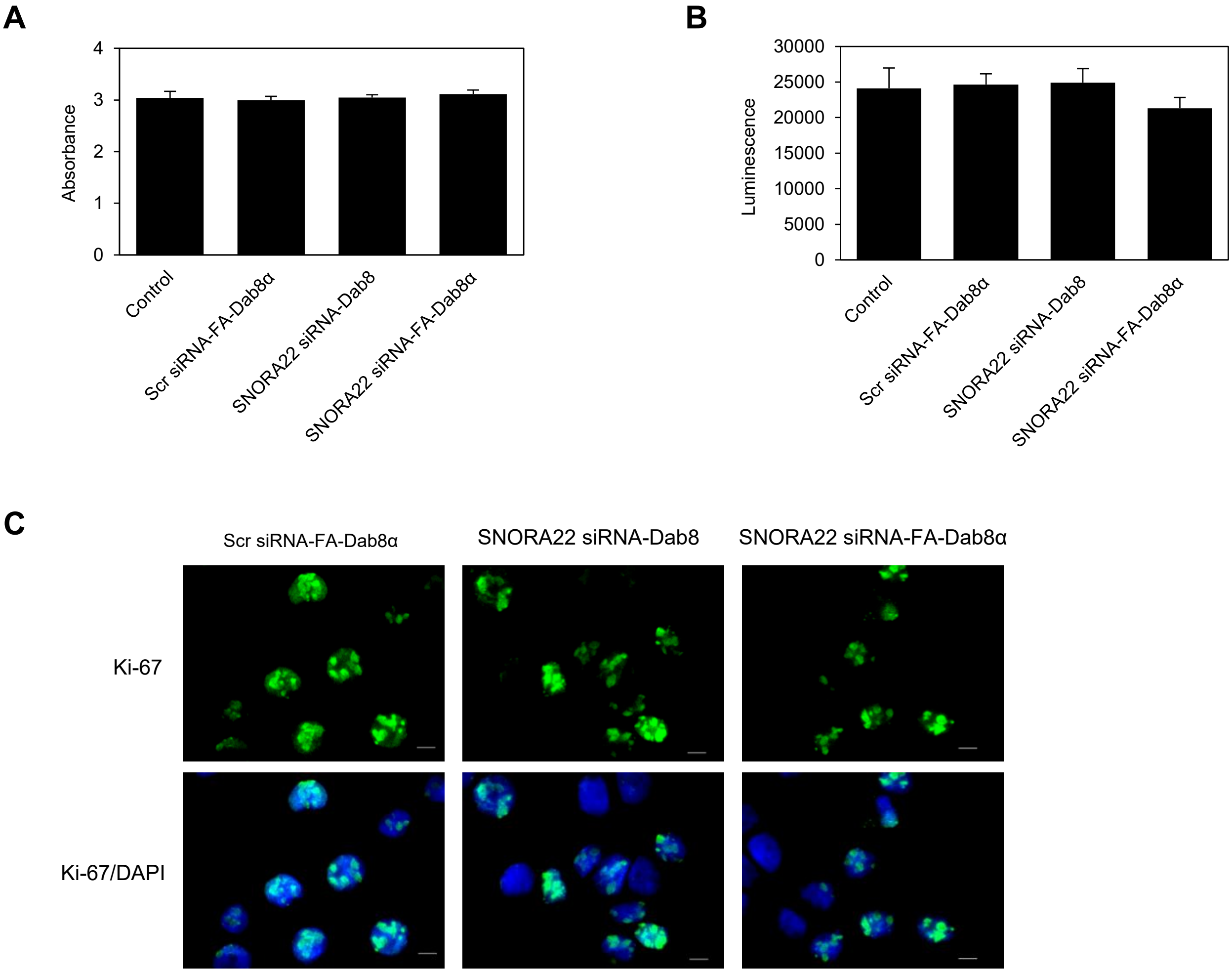

Supplementary Figure 5. Effects of *SNORA22* knockdown on cell viability, apoptosis, and cell proliferation in S2-013 cells *in vitro*.

(A) Cell viability evaluated by the MTT assay in S2-013 cells following treatment with untreated control, scrambled control siRNA-FA-Dab8α, chemically modified SNORA22 siRNA-FA-Dab8α, and chemically modified SNORA22 siRNA-Dab8. Data are presented as the mean ± SD of three independent experiments.

(B) Apoptosis rates evaluated by the Caspase 3/7 activity assay in S2-013 cells treated with untreated control, scrambled control siRNA-FA-Dab8α, chemically modified SNORA22 siRNA-FA-Dab8α, and chemically modified SNORA22 siRNA-Dab8. Luminescence values representing Caspase 3/7 activity are presented as the mean ± SD of three independent experiments.

(C) Representative confocal immunofluorescence images of Ki-67 staining (green) and nuclear counterstaining with DAPI (blue) in S2-013 cells treated with scrambled control siRNA-FA-Dab8α, chemically modified SNORA22 siRNA-FA-Dab8α, or chemically modified SNORA22 siRNA-Dab8. Scale bars, 10 μm.

Supplementary Figure 6

A

a **b**

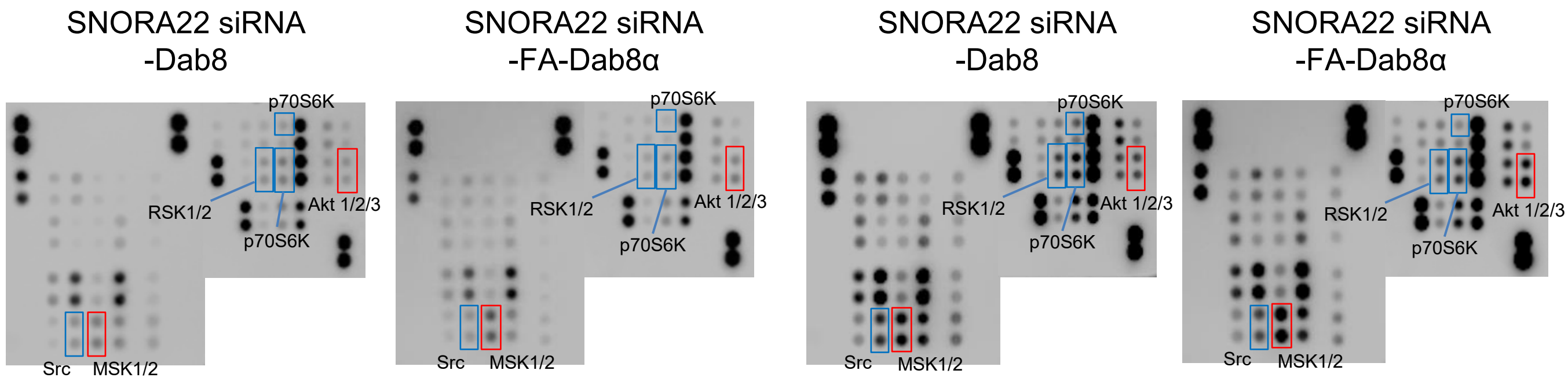

B

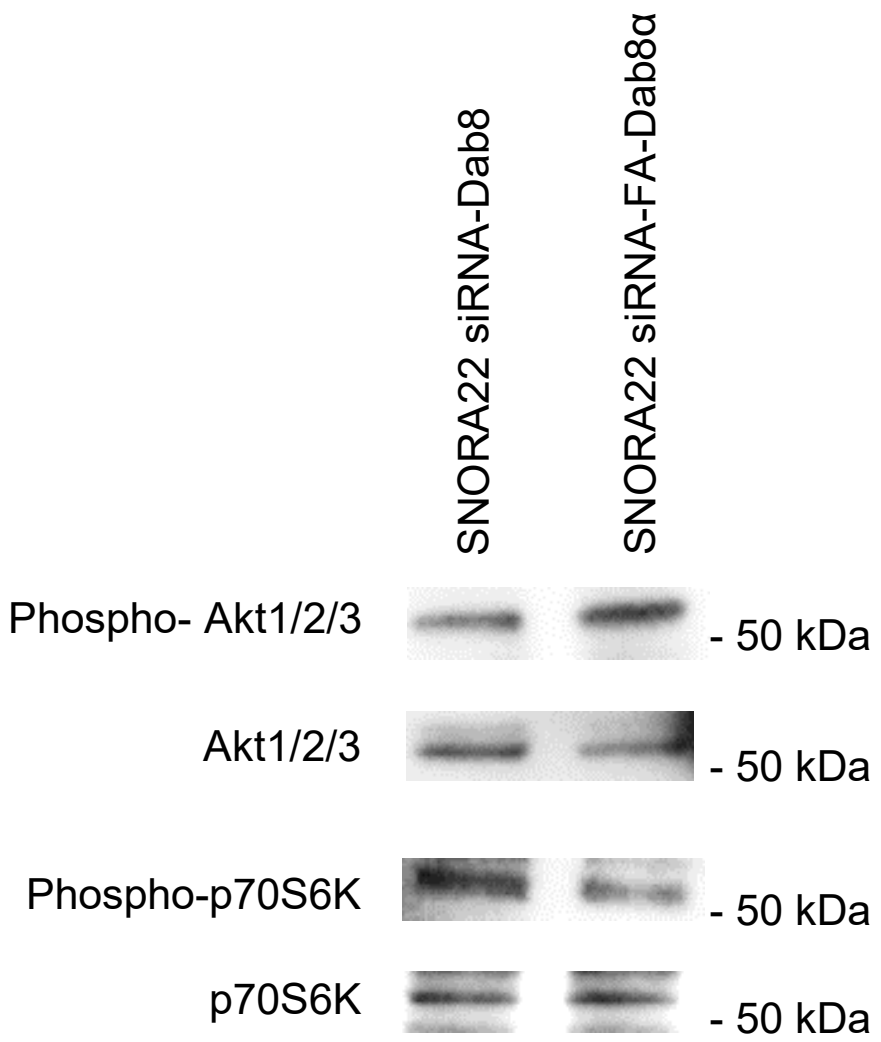

Supplementary Figure 6. Phospho-protein array analysis showing downstream signaling alterations following *SNORA22* knockdown.

(A) Representative human phospho-protein array profiling of S2-013 cells treated with the chemically modified SNORA22 siRNA-FA-Dab8α complex compared with those treated with the chemically modified SNORA22 siRNA-Dab8 complex. Images with shorter (a) and longer (b) exposure times are shown. Red boxes indicate increased phosphorylation/activity, whereas blue boxes indicate decreased phosphorylation/activity in cells treated with the SNORA22 siRNA-FA-Dab8α complex.

(B) Western blot analysis validating the phosphorylation levels of p70S6K and Akt1/2/3 in S2-013 cells treated with the chemically modified SNORA22 siRNA-FA-Dab8α complex or the chemically modified SNORA22 siRNA-Dab8 complex.

Supplementary Figure 7

Scr siRNA-FA-Dab8α

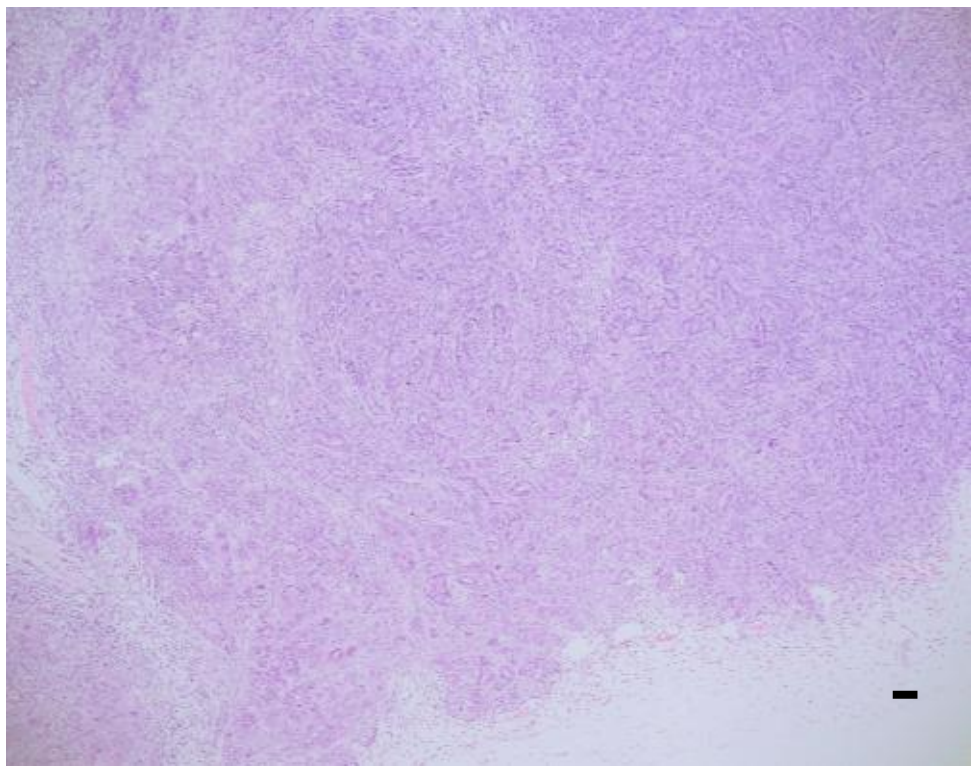

SNORA22 siRNA-Dab8

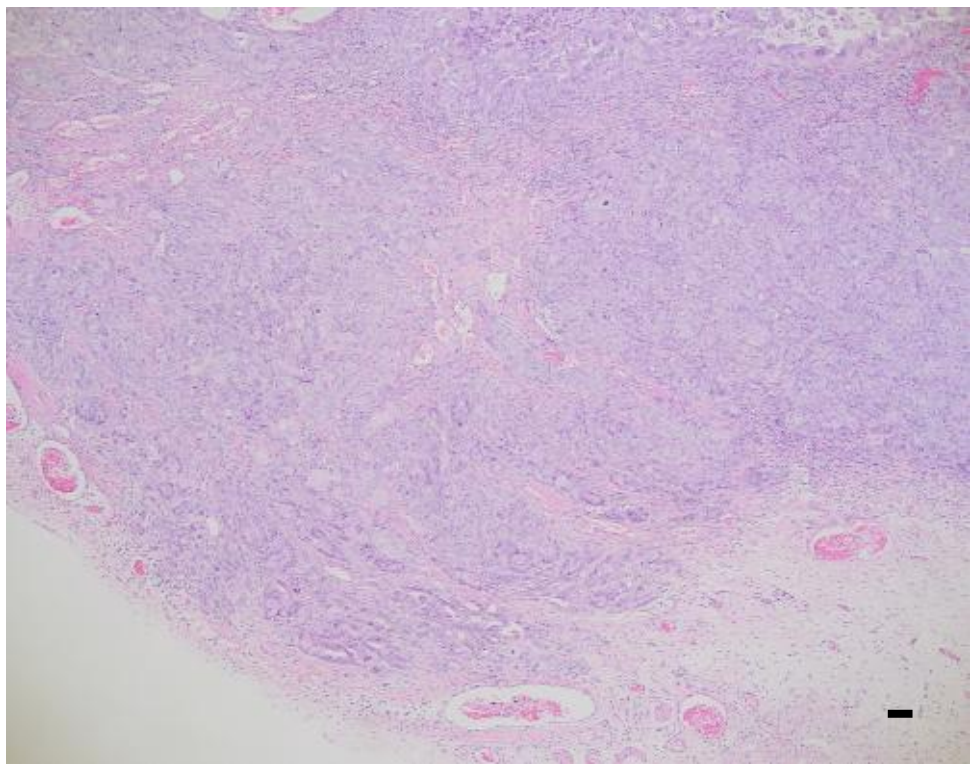

SNORA22 siRNA-FA-Dab8α

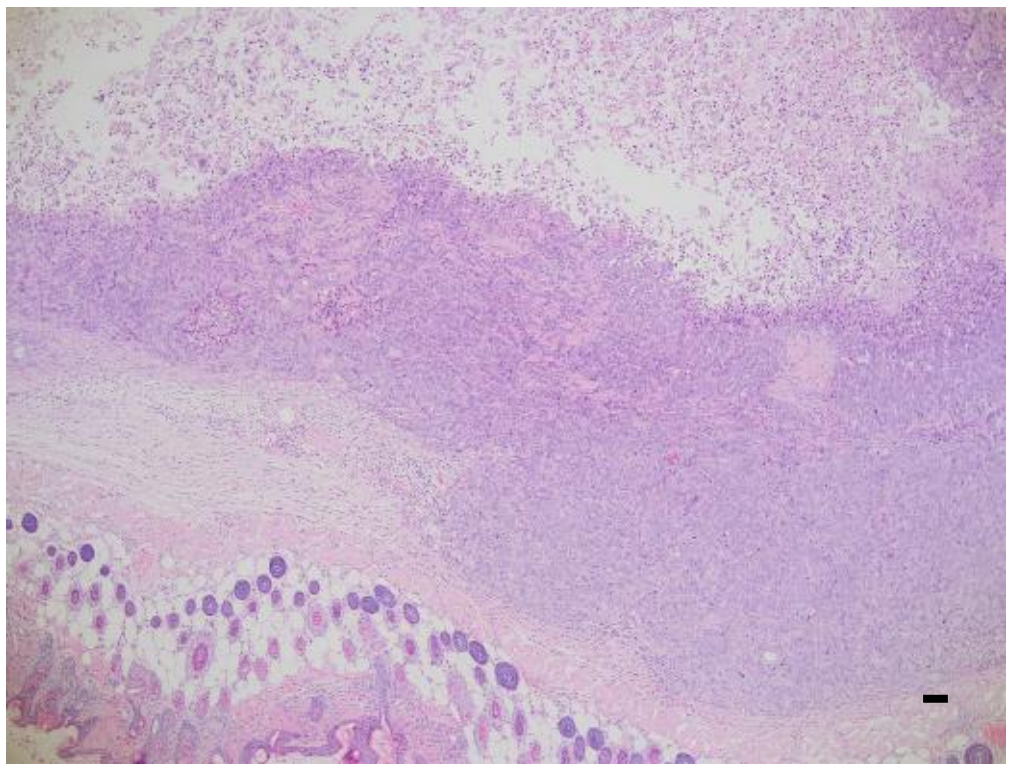

GP

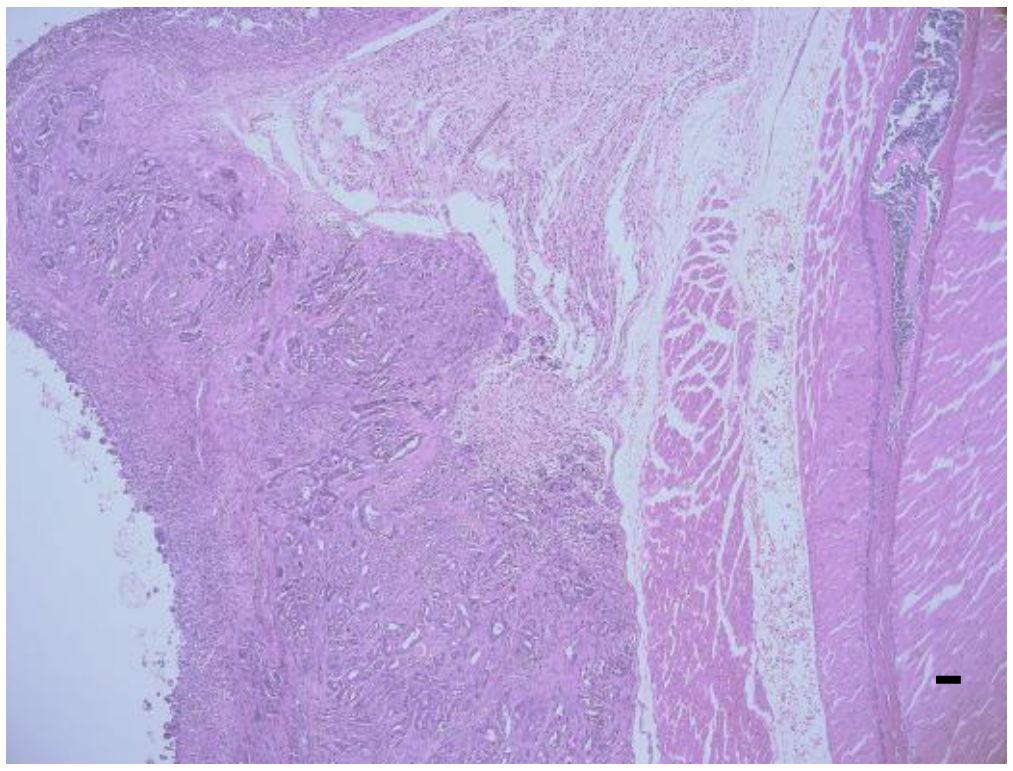

Supplementary Figure 7. Low-magnification histological evaluation of tumor xenografts.
Representative low-magnification H&E-stained images of tumor xenografts from each treatment group (scrambled control siRNA–FA-Dab8α, chemically modified SNORA22 siRNA-Dab8, chemically modified SNORA22 siRNA-FA-Dab8α, and gemcitabine plus paclitaxel [GP]), providing lower-power views of the areas evaluated in Fig. 8B. Scale bars, 50 μm.
